# Genes related to inflammation and bone loss process in periodontitis suggested by bioinformatics methods

**DOI:** 10.1186/s12903-015-0086-7

**Published:** 2015-09-04

**Authors:** Liang Song, Jueqi Yao, Zhijing He, Bin Xu

**Affiliations:** Department of Stomatology, The Fifth People’s Hospital of Shanghai, Fudan University, No.128, Ruili Rd, Minhang District, Shanghai, 200240 China; Department of Endodontics, Shanghai Oral Disease Prevention and Cure Center, Shanghai, 200031 China; Department of Stomatology, The Second Xiangya Hospital of Central South University, Changsha, 410011 Hunan Province China

## Abstract

****Background**:**

Despite of numerous studies on periodontitis, the mechanism underlying the progression of periodontitis still remains largely unknown. This study aimed to have an expression profiling comparison between periodontitis and normal control and to identify more candidate genes involved in periodontitis and to gain more insights into the molecular mechanisms of periodontitis progression.

**Methods:**

The gene expression profile of GSE16134, comprising 241 gingival tissue specimens and 69 healthy samples as control which were obtained from 120 systemically healthy patients with periodontitis (65 with chronic and 55 with aggressive periodontitis), was downloaded from the Gene Expression Omnibus (GEO) database. Differentially expressed genes (DEGs) in periodontitis samples were screened using the limma package in R compared with control samples. Gene Ontology (GO) and pathway enrichment analysis upon the DEGs were carried out using Hypergeometric Distribution test. Protein-protein interaction (PPI) network of the DEGs was constructed using Cytoscape, followed by module selection from the PPI network using MCODE plugin. Moreover, transcription factors (TFs) of these DEGs were identified based on TRANSFAC database and then a regulatory network was constructed.

**Results:**

Totally, 762 DEGs (507 up- and 255 down-regulated) in periodontitis samples were identified. DEGs were enriched in different GO terms and pathways, such as immune system process, cell activation biological processes, cytokine-cytokine receptor interaction, and metabolic pathways. Cathepsin S (*CTSS*) and pleckstrin (*PLEK*) were the hub proteins in the PPI network and 3 significant modules were selected. Moreover, 19 TFs were identified including interferon regulatory factor 8 (IRF8), and FBJ murine osteosarcoma viral oncogene homolog B (FOSB).

**Conclusion:**

This study identified genes (*CTSS*, *PLEK*, *IRF-8*, *PTGS2*, and *FOSB*) that may be involved in the development and progression of periodontitis.

**Electronic supplementary material:**

The online version of this article (doi:10.1186/s12903-015-0086-7) contains supplementary material, which is available to authorized users.

## Background

Periodontitis is a chronic inflammatory disease involving interactions between complex microbial biofilms, many cell populations and inflammatory mediators, leading to the destruction of the tooth-supporting structures like the periodontal ligament and the alveolar bone [1]. Besides being a common cause of tooth loss, severe periodontitis (about 8.5 % of patients) can detrimentally affect systemic health, as it can increase the patients’ risk for diabetes, atherosclerosis, rheumatoid arthritis, and adverse pregnancy outcomes [2–4]. Two major clinical entities of periodontitis are currently recognized: chronic periodontitis, which is more common, and aggressive periodontitis, a clinically challenging entity featured by an early onset and a rapid progression [5]. The underlying etiology of both the two forms has not been fully elucidated. Therefore, gaining further insights into the molecular mechanisms of periodontitis will be of great significance for the treatment of periodontitis.

Previous studies have demonstrated that factors that may determine the presence and rate of progression of periodontitis are complex, which can be defined as the interplay of numerous parameters acting simultaneously and unpredictably [1]. For instance, the tooth-associated microbial biofilm or dental plaque is essential but not sufficient to induce periodontitis. The host inflammatory response to the microbial challenge can finally cause the destruction of the periodontium [6]. Inflammation and bone loss are hallmarks of periodontal disease [7] and accumulated evidence demonstrates that a number of mediators are involved in these processes [8]. Cochran et al. had reported that the reduction of inflammation and attenuation of the host’s immune reaction to the microbial plaque could lead to a decrease in the ratio of nuclear factor-kappa B ligand (RANKL)/osteoprotegerin (OPG) and a decrease in associated bone loss [7]. Besides, a study has reviewed that cytokines such as interleukin-1 (IL-1) and tumor necrosis factor (TNF) are a significant and integral component of the host reaction to periodontal infection [8]. In addition, secreted IL-8 induced by multiple stimuli like live bacteria and proinflammatory cytokines is associated with the inflammation and invasiveness of periodontitis [9]. Despite of numerous investigations on periodontitis, the mechanism still remains largely unknown.

Using the same gene expression profile, Stoecklin et al. identified specific miRNAs (has-miR-210 and hsa-miR-185) and their target genes in gingival tissues [10]. In addition, Kebschull et al. found that small differences in gene expression and the highly variable classifier performance suggested limited dissimilarities between established chronic periodontitis and aggressive periodontitis lesions [11]. We sought to have an expression profiling comparison between periodontitis (chronic periodontitis and aggressive periodontitis collectively) and normal control, identifying more candidate genes involved in both chronic and aggressive periodontitis and to gain more insights into the molecular mechanisms of periodontitis progression.

## Methods

### Microarray data and data preprocessing

The gene expression profile data of GSE16134 [11] was downloaded from the Gene Expression Omnibus (GEO) in NCBI (http://www.ncbi.nlm.nih.gov/geo/) based on the platform of GPL570 [HG-U133_Plus_2] Affymetrix Human Genome U133 Plus 2.0 Array. This dataset included 241 ‘diseased’ gingival tissue specimens (bleeding on probing, probing depth ≥ 4 mm, and clinical attachment loss ≥ 3 mm) and 69 ‘healthy’ gingival tissue samples (no bleeding on probing, probing depth ≤ 4 mm, and clinical attachment loss ≤ 4 mm), obtained from 120 systemically healthy, non-smoking individuals with moderate/severe periodontitis (65 with chronic and 55 with aggressive periodontitis), as previously described [11, 12]. The 120 patients undergoing periodontal surgery contributed with a minimum of two interproximal gingival papillae from a maxillary posterior and when available, a ‘healthy’ papilla was obtained. In the present study, samples from chronic and aggressive periodontitis patients were taken together as one group, designed as periodontitis samples, and were compared with the 69 ‘healthy’ gingival tissue samples as controls in the following analysis.

The downloaded profile had been preprocessed which was carried out with background correction, log_2_ transformation, and quantile normalization using the RMA (Robust Multi-array Analysis) method of affy package in R [13]. In this study, those probes hybridizing to the same gene were normalized using the preprocessCore package [14]. The gene expression matrix of specimens was received and was used to the follow-up analysis.

### Screening of DEGs

The DEGs in periodontitis samples were screened compared with control samples using the Linear Models for Microarray data (limma) package in R [15]. False discovery rate (FDR) [16] was applied for multiple testing correction using Benjamini and Hochberg method [17]. Threshold for the DEGs were set as FDR < 0.05 and |log_2_ FC (fold change)| ≥ 0.58.

### Function and pathway enrichment analysis of DEGs

In order to investigate the periodontitis progression on the perspective of functional level, Gene Ontology (GO) and pathway enrichment analysis of the identified DEGs were performed in this study. GO categories such as biological process (BP), molecular function (MF), and cellular component (CC) upon the identified DEGs were enriched from GO databases using the Hypergeometric Distribution test [18]. Also, the pathways that the DEGs involved in were enriched using the Hypergeometric Distribution test from the KEGG (Kyoto Encyclopedia of Genes and Genomes) database [19], which had not been used by Kebschull et al. [12]. The p-value < 0.05 was chosen as the threshold.

### PPI network construction of DEGs and modules selection

The STRING (Search Tool for the Retrieval of Interacting Genes/Proteins) database, which is a database of known and predicted protein interactions [20], was used to select the interactions among the selected DEGs in this study, which were not used in the study of Kebschull et al. [12]. The PPI network was constructed by functional links between proteins that are experimentally derived, as well as links predicted by co-expression analysis and text mining, or PPIs which had related records in the database. Genes included in the PPI network were all DEGs. Besides, the combined score ≥ 0.4 were chosen for the PPI network construction. Cytoscape software [21] was used to visualize the constructed network while MCODE [22] was used to select significant modules from the PPI network (Parameters: Degree cutoff: 2, Node score cutoff: 0.2, K-core: 2, Max. depth: 100). Furthermore, topological analysis of the PPI network was performed and node degrees of these DEGs were analyzed.

### Function annotation of the DEGs and construction of regulatory network

To identify the DEGs which had the transcriptional regulatory functions, the identified DEGs in this study were analyzed using the TRANSFAC database (http://www.gene-regulation.com/pub/databases.html) [23], a database comprising data of transcription factors (TFs), their target genes and regulatory binding sites. Moreover, a regulatory network based on the identified TFs and their target DEGs was constructed. While in the study of Kebschull et al. [12], TRANSFAC database had not been used.

## Results

### Data preprocessing and DEGs screening

In the original analysis of Kebschull et al. [12], a total of 248 differentially regulated probes were identified at an absolute fold change of ≥1.19, and 30 overexpressed only one under-expressed probe by an absolute change of >1.5 fold were identified in aggressive periodontitis lesions compared with chronic periodontitis lesions. However, in this study, after data preprocessing, 20,303 genes were mapped to the probes. Compared with the control samples, a total of 762 DEGs were identified in the periodontitis samples (Additional file 1: Table S1), including 507 up-regulated genes and 255 down-regulated genes.

### GO and pathway enrichment analysis of DEGs

Functional and pathway enrichment analysis indicated that up-regulated DEGs and down-regulated DEGs in the periodontitis samples were significantly enriched in different GO terms and KEGG pathways (Tables 1 and 2). Top 5 GO terms of up- and down-regulated genes were shown in Table 1, respectively. The up-regulated genes were involved in different GO terms such as cell activation, activation of immune response, chemokine activity, and antigen binding (Table 1A). While the down-regulated genes were associated with the GO terms like skin development, epidermal cell differentiation, intermediate filament, and structural molecule activity (Table 1B). On the other hand, top 10 pathways of up- and down-regulated genes were shown in Table 2, respectively. The pathway enrichment analysis showed that the up-regulated genes were mainly involved in staphylococcus aureus infection and cytokine-cytokine receptor interaction (Table 2A), while the down-regulate genes were mainly associated with the pathways such as metabolic pathways, phagosome, and melanogenesis pathways (Table 2B).

**Table 1:** The functional analysis of the DEGs

Category	GO ID	Name	Count	*p*-value
A: the top 5 GO terms of the up-regulated DEGs				
BP	GO:0001775	cell activation	68	<1.00E-16
BP	GO:0002253	activation of immune response	48	<1.00E-16
BP	GO:0002376	immune system process	165	<1.00E-16
BP	GO:0002682	regulation of immune system process	94	<1.00E-16
BP	GO:0002684	positive regulation of immune system process	73	<1.00E-16
CC	GO:0005576	extracellular region	138	<1.00E-16
CC	GO:0005615	extracellular space	70	<1.00E-16
CC	GO:0044421	extracellular region part	86	<1.00E-16
CC	GO:0071944	cell periphery	191	2.64E-14
CC	GO:0005886	plasma membrane	186	1.49E-13
MF	GO:0008009	chemokine activity	10	2.35E-07
MF	GO:0003823	antigen binding	12	4.64E-07
MF	GO:0032403	protein complex binding	26	4.85E-07
MF	GO:0042379	chemokine receptor binding	10	1.11E-06
MF	GO:0005178	integrin binding	12	1.41E-06
B: the top 5 GO terms of the down-regulated DEGs				
BP	GO:0043588	skin development	311	2.33E-14
BP	GO:0008544	epidermis development	280	1.18E-12
BP	GO:0030216	keratinocyte differentiation	108	1.09E-09
BP	GO:0009913	epidermal cell differentiation	154	1.01E-08
BP	GO:0009888	tissue development	1479	2.09E-08
CC	GO:0030057	desmosome	22	5.73E-06
CC	GO:0005882	intermediate filament	191	2.33E-05
CC	GO:0045111	intermediate filament cytoskeleton	231	2.76E-05
CC	GO:0045095	keratin filament	93	0.00096928
CC	GO:0005911	cell-cell junction	297	0.00108577
MF	GO:0005198	structural molecule activity	627	2.89E-05
MF	GO:0005200	structural constituent of cytoskeleton	94	0.00104197
MF	GO:0016755	transferase activity, transferring amino-acyl groups	24	0.003031659
MF	GO:0016702	oxidoreductase activity, acting on single donors with incorporation of molecular oxygen, incorporation of two atoms of oxygen	25	0.003414279
MF	GO:0016701	oxidoreductase activity, acting on single donors with incorporation of molecular oxygen	26	0.003825148

*MF* molecular function, *BP* biological process, *CC* cell component

**Table 2:** The pathway enrichment analysis of the DEGs

KEGG ID	Name	Count	*p*-value
A: the top 10 enriched pathways of the up-regulated DEGs			
5150	Staphylococcus aureus infection	55	4.40E-12
5144	Malaria	51	2.12E-09
4060	Cytokine-cytokine receptor interaction	265	2.39E-08
4141	Protein processing in endoplasmic reticulum	165	5.11E-07
4640	Hematopoietic cell lineage	88	5.53E-07
5323	Rheumatoid arthritis	91	8.66E-07
5140	Leishmaniasis	72	1.64E-06
4670	Leukocyte transendothelial migration	116	4.30E-06
4514	Cell adhesion molecules (CAMs)	133	6.18E-06
4062	Chemokine signaling pathway	189	1.71E-05
B: the top 10 enriched pathways of the down-regulated DEGs			
590	Arachidonic acid metabolism	59	0.002776062
980	Metabolism of xenobiotics by cytochrome P450	71	0.005420335
982	Drug metabolism - cytochrome P450	73	0.005982341
350	Tyrosine metabolism	41	0.007842248
1100	Metabolic pathways	1130	0.012102847
5144	Malaria	51	0.014274311
5014	Amyotrophic lateral sclerosis (ALS)	53	0.015832167
4145	Phagosome	153	0.018215247
4916	Melanogenesis	101	0.018240082
563	Glycosylphosphatidylinositol (GPI)-anchor biosynthesis	25	0.025692313
830	Retinol metabolism	64	0.026060907
591	Linoleic acid metabolism	30	0.036083866

### PPI network construction and modules selection

**Fig. 1:**
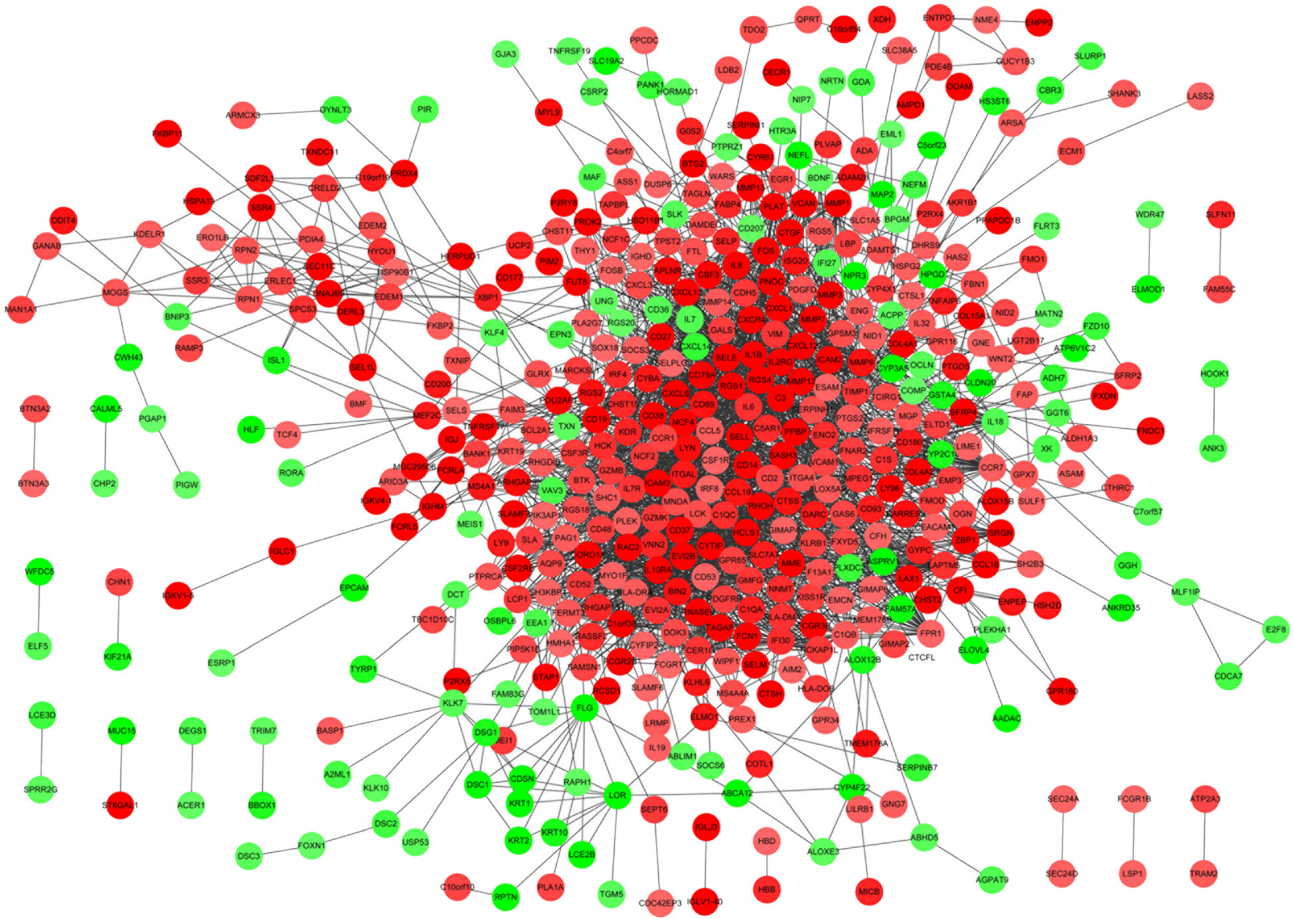
Protein-protein interaction (PPI) network of the differentially expressed genes (DEGs). Red nodes stand for the up-regulated DEGs while green nodes stand for the down-regulated genes

**Fig. 2:**
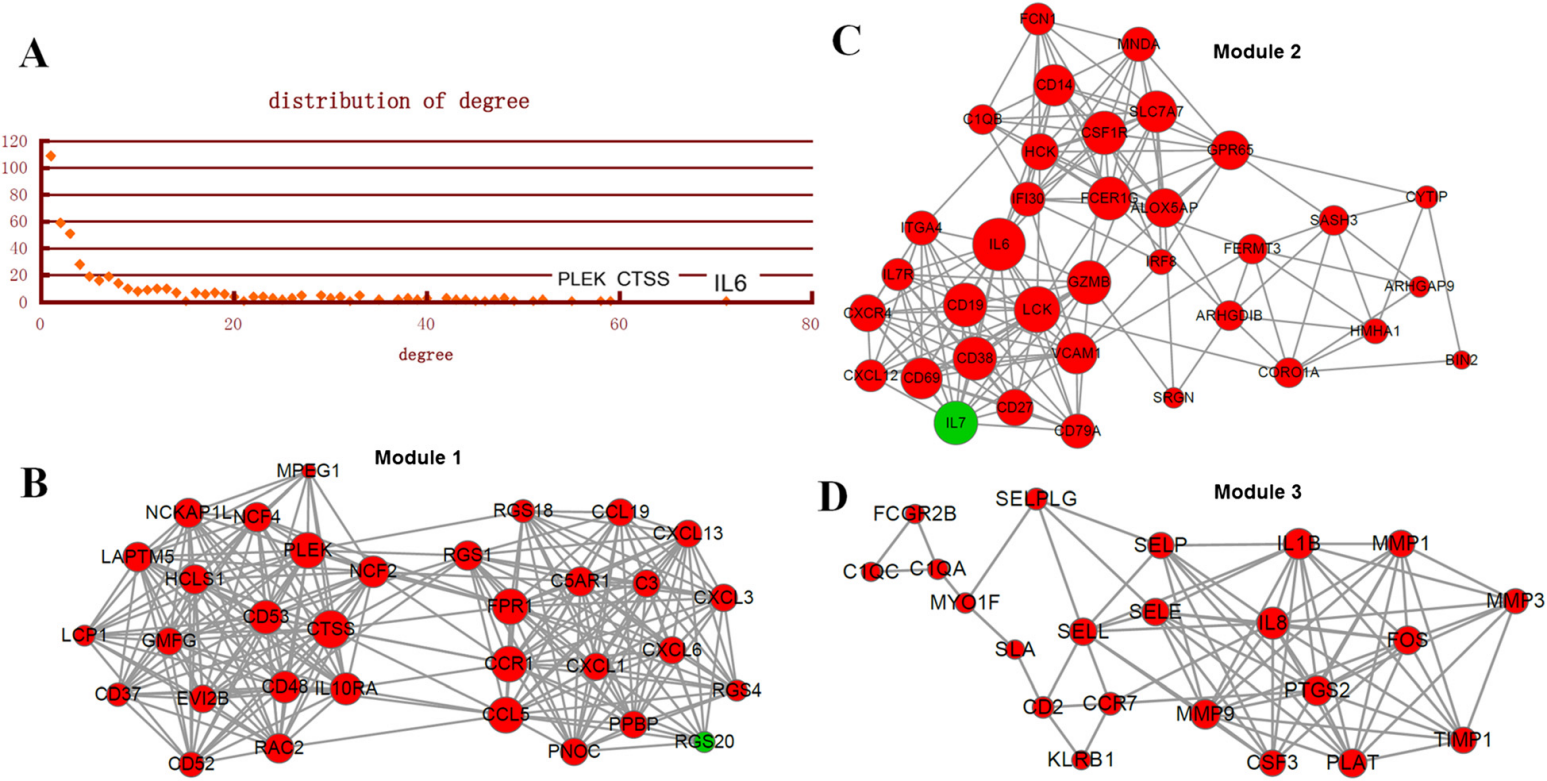
Analysis of the PPI network.A, topological analysis of the degrees of the DEGs in the PPI network. Horizontal axis stands for the degree of one DEG and vertical axis stands for the number of nodes. B, module 1 of DEGs from PPI network. C, module 2 of DEGs from PPI network. D, module 3 of DEGs from PPI network. Red nodes stand for the up-regulated DEGs while green nodes stand for the down-regulated genes. The size of one node is proportional to the degree of this gene

The PPI network upon the DEGs was shown in Fig. 1. The results of topological analysis showed that interleukin-6 (*IL-6*), cathepsin S (*CTSS*), and pleckstrin (*PLEK*) were the hub proteins in the PPI network which had higher node degrees (Fig. 2a). In addition, 3 significant modules were selected from the PPI network (Fig. 2). From the results, we found that most of the genes enriched in the 3 modules were up-regulated. In particular, the top 5 genes with higher node degrees in module 1 were *CTSS* (degree = 20), chemokine (C-C motif) ligand 5 (*CCL5)* (degree = 19), *PLEK* (degree = 19), chemokine (C-C motif) receptor 1 (*CCR1*) (degree=19), and formyl peptide receptor 1 (*FPR1*) (degree=19) (Fig. 2b, Table 3). The top 5 genes with higher node degrees in module 2 were *IL6* (degree = 17), lymphocyte-specific protein tyrosine kinase (*LCK*) (degree = 14), Fc fragment of IgE, high affinity I, receptor for; gamma polypeptide (*FCER1G*) (degree = 13), *CD19* (degree = 13), and colony stimulating factor 1 receptor (*CSF1R*) (degree = 13) (Fig. 2c, Table 3). Additionally, the top 5 genes with higher node degrees in module 3 were *IL8* (degree = 13), *IL1B* (degree = 12), *MMP9* (degree = 11), prostaglandin-endoperoxide synthase 2 (*PTGS2*) (degree=11), and plasminogen activator, tissue (*PLAT*) (degree=10) (Fig. 2d, Table 3).

**Table 3:** The top 5 DEGs with higher degrees in the selected modules

Module	Gene	Expression changes	Degree
module 1	CTSS	up	20
	CCL5	up	19
	PLEK	up	19
	CCR1	up	19
	FPR1	up	19
Module 2	IL6	up	17
	LCK	up	14
	FCER1G	up	13
	CD19	up	13
	CSF1R	up	13
Module 3	IL8	up	13
	IL1B	up	12
	MMP9	up	11
	PTGS2	up	11
	PLAT	up	10

### Regulatory network construction

**Fig. 3:**
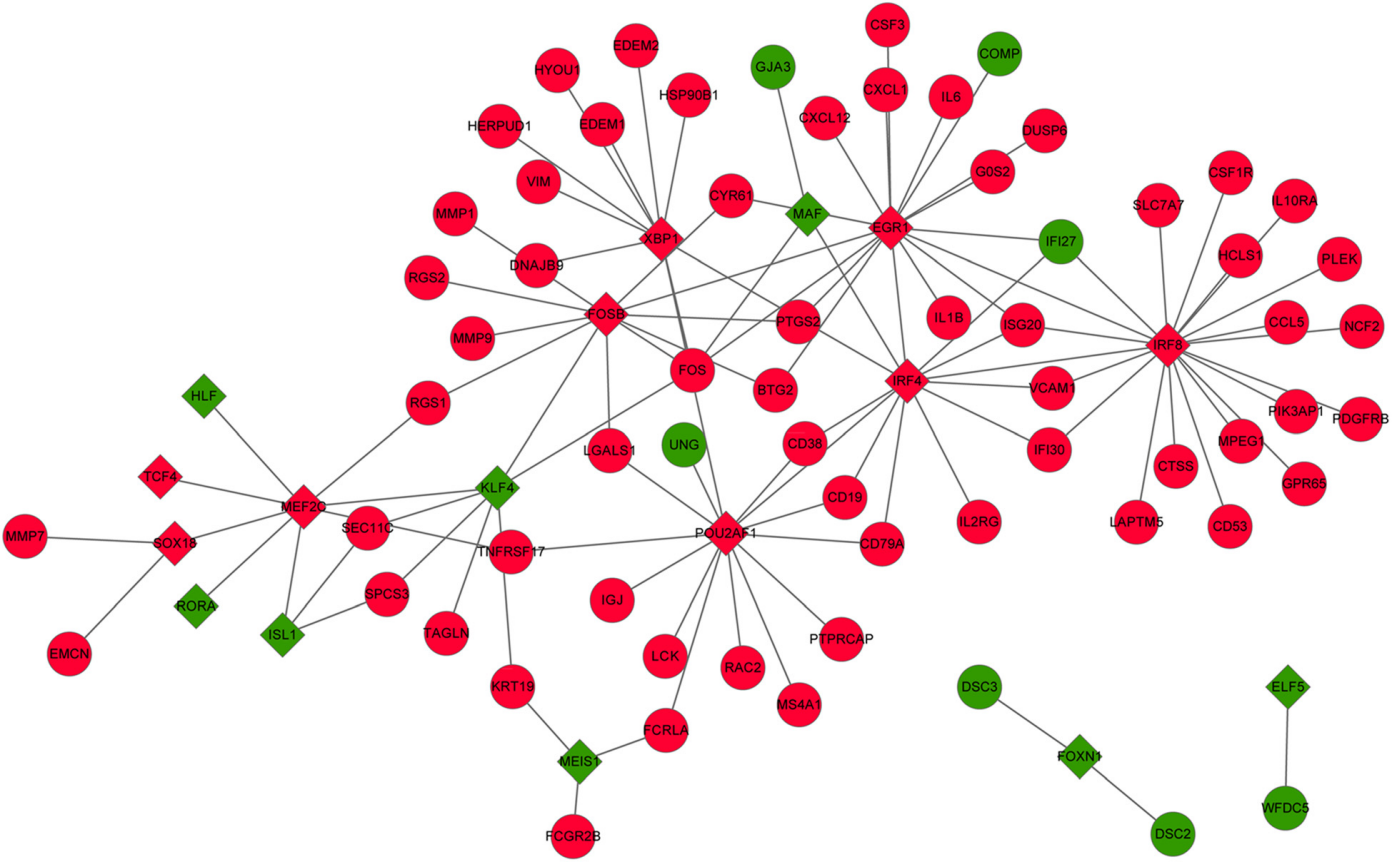
Regulatory network of the transcription factors-DEGs. Diamond represents the transcription factor while the circle represents the DEG. Red color stands for up-regulated expression while green color stands for down-regulated expression

A total of 9 TFs were identified from the up-regulated DEGs, such as interferon regulatory factor 4 (IRF4), IRF8, and FBJ murine osteosarcoma viral oncogene homolog B (FOSB). Besides, 10 TFs from the down-regulated DEGs were selected, such as Kruppel-like factor 4 (KLF4), v-maf avian musculoaponeurotic fibrosarcoma oncogene homolog (MAF), and Meis homeobox 1 (MEIS1). The regulatory network of these TFs and their target genes was shown in Fig. 3. From the results, we found that several DEGs with higher degrees in module 1 could be regulated by IRF8, for example, *CTSS*, *CCL5*, and *PLEK*.

## Discussion

In this study, we used the microarray data to select genes associated with periodontitis. Totally, 762 DEGs in the periodontitis samples were identified compared with the control samples. The up-regulated genes were mainly enriched in the GO terms like cell activation and activation of immune response, as well as the pathways such as staphylococcus aureus infection and cytokine-cytokine receptor interaction. The down-regulated genes were mainly linked to tissue development and metabolism pathways. *CTSS*, *PLEK*, *LCK*, and *PTGS2* were identified to be hub proteins in the PPI network or in the selected module. Besides, 9 TFs and 10 TFs were selected from the up-regulated genes and down-regulated genes respectively, for example, IRF4, IRF8, and FOSB.

Our results showed that 20,303 genes were mapped to the probes. Compared with the healthy samples, a total of 762 DEGs were identified in the periodontitis samples, including 507 up-regulated genes (FDR< 0.05 and log_2_ FC ≥ 0.58) and 255 down-regulated genes (FDR< 0.05 and log_2_ FC < −0.58). While, Kebschull et al. identified a total of 248 differentially regulated probes at an absolute fold change of ≥1.19 [12]. They reported 30 overexpressed and only one under-expressed probe by an absolute change of >1.5 fold in aggressive periodontitis lesions compared with chronic periodontitis lesions. Besides, they found that 9258 probes were differentially expressed when compared the ‘diseased’ tissues with ‘healthy’ gingival tissues. Collectively, the results showed that we identified distinct genetic features in periodontitis samples using different screening methods with different thresholds.

In this study, we found that DEGs in periodontitis samples were mainly enriched in different GO terms and pathways, such as cell activation, activation of immune response, staphylococcus aureus infection and cytokine-cytokine receptor interaction, using KEGG database which were not used by Kebschull et al. [12]. In their investigations, gene set enrichment analysis was performed and gene sets linked to apoptosis, immune response were enriched in aggressive periodontitis lesions, while genes sets linked to cellular metabolism and epithelial integrity were enriched in chronic periodontitis lesions [12]. In a susceptible host, persistence of bacteria pathogens such as *Porphyromonas gingivalis* results in aberrant and extended inflammation and subsequent destruction of the tooth-supporting structures [24]. The immune cells such as antigen presenting cells (APC) initially responding to the challenge by bacteria pathogens, including *Porphyromonas gingivalis*, poised strategically along portals of entry [25]. After recognition of pathogen associated molecular patterns (PAMPs) via pattern recognition receptors (eg, toll like receptors [TLRs]), innate immune cells start responses aiming to clear the inciting agent [26]. Moutsopoulos et al. had showed that *Porphyromonas gingivalis* could promote T helper cell 17 (Th17) inducing pathways in chronic periodontitis [24]. Thus, the enrichment results identified in our study was in accordance with the previous studies.

CTSS is a lysosomal cysteine proteinase that may participate in the degradation of antigenic proteins to peptides for presentation on MHC class II molecules [27]. Deficiency of CTSS induces a high bone turnover and then leading to the less dense bone [28]. Mogi et al. demonstrated that the expression level of the key bone degradation enzyme cathepsin K (another member of family proteins) in gingival crevicular fluid tissues of periodontitis patients was higher than that in normal tissues [29]. Besides, IRF8 can specifically bind to the upstream regulatory region of type I interferon (IFN). Zhao et al. had demonstrated that IRF-8 was a regulator for osteoclastogenesis in bone metabolism [30]. Soft tissue destruction and bone degradation were often found in periodontitis [31]. Moreover, a study revealed that CTSS had the binding site for transcription factor IRF1, and combination of IRF8 and IRF1 could promote the CTSS expression [32]. In the present study, CTSS was a hub protein in the PPI network and could be regulate by IRF8 in the regulatory network. In the context, we suggested that *CTSS* might play an essential role in bone loss involved in periodontitis progression by interacting with *IRF8*.

On the other hand, PLEK is a major substrate of protein kinase C in platelets and leukocytes and appears to play an important role in exocytosis through a currently unknown mechanism [33]. Ding et al. proved that the phosphorylated PLEK increased the secretion of proinflammatory cytokine in mononuclear phagocytes [34]. Besides, Ueki et al. had showed that the secreted monocytes activated by bacterial in gingival crevicular fluid was associated with periodontitis [35]. On the other hand, IRF8 can specifically bind to the upstream regulatory region of IFN [36]. Additionally, Bar-Or et al. showed that B cells could exhibit abnormal proinflammatory cytokine responses (such as exaggerated production of TNF) when activated in the context of the Th1 cytokine IFN [37]. In this study, the results showed that *PLEK* was a hub protein in the PPI network and could be regulated by IRF8 in the regulatory network. Therefore, we speculated that *PLEK* might contribute to the periodontitis progression via interacting with *IRF-8*.

PTGS2 is an isozyme of PTGS which is the key enzyme in prostaglandin biosynthesis, and acts both as a dioxygenase and as a peroxidase [38]. The study of Zhang et al. had demonstrated that there was a hypermethylation pattern of the promoter in connection with a lower level of PTGS2 transcription in the inflamed tissues in chronic periodontitis [39]. On the other hand, FOSB is one member of the Fos gene family which encodes leucine zipper proteins that can dimerize with proteins of the JUN family [40]. T cell receptor (TCR)-driven early gene expression is controlled by numerous key transcription factors such as FOSB [41]. Additionally, Sreeramkumar et al. had reported that PTGS2 was transcriptionally up-regulated in T cells during TCR/CD3 triggering and that it behaved as an early inducible gene in the T cell activation process [42]. Moreover, Chen et al. had demonstrated that costimulatory double signals from CD28 and TCR were required for optimal expression of receptor activator of nuclear factor-κB ligand (RANKL) in periodontal tissues [43]. In the present study, the results showed that *PTGS2* was involved in module 3 and could be regulated by FOSB in the regulatory network. Thus, we suggested that *PTGS2* might play a critical role in periodontitis progression involving in TCR signaling pathway via interacting with *FOSB.*

## Conclusion

In conclusion, this study identified several genes (*CTSS*, *PLEK*, *IRF-8*, *PTGS2* and FOSB) that involved in the development and progression of periodontitis. *CTSS* may play an essential role in bone loss associated with periodontitis by interacting with *IRF8.* Besides, *PLEK* may contribute to the periodontitis progression via interacting with *IRF-8*. In addition, *PTGS2* may play a critical role in periodontitis progression involving in TCR signaling pathway via interacting with *FOSB.* Our study may provide theoretical basis for the future investigations of periodontitis. However, further experimental studies are still needed to confirm our results.

## Additional file

Additional file 1: Table S1. All the differentially expressed genes (DEGs) in periodontitis samples with their corresponding modules (DOCX 102 kb)

## Abbreviations

APC: Antigen presenting cells
BP: Biological processes, CTSS, Cathepsin S, CC, Cellular component
DEGs: Differentially expressed genes, IRF4, Factor 4, FDR, False discovery rate, GO, Gene Ontology
IL-6: Interleukin-6
IL-8: Interleukin-8, KEGG, Kyoto Encyclopedia of Genes and Genomes
KLF4: Kruppel-like factor 4
MMP: Matrix metalloproteinase, MEIS1, Meis homeobox 1
MF: Molecular functions
MAF: Musculoaponeurotic fibrosarcoma oncogene homolog, PLEK, Pleckstrin
PAMPs: Pathogen associated molecular patterns
PPI: Protein-protein interaction
STRING: Search Tool for the Retrieval of Interacting Genes/Proteins)
Th17: T helper cell 17
TLR: Toll like receptors, TFs, Transcription factors
