# Supplementary material for: Genes related to inflammation and bone loss process in periodontitis suggested by bioinformatics methods
Source: BMC Oral Health. 2015 Sep 4;15:105. doi: 10.1186/s12903-015-0086-7 (PMC4559289; doi:10.1186/s12903-015-0086-7)
Supplement: Additional file 1: Table S1. — All the differentially expressed genes (DEGs) in periodontitis samples with their corresponding modules (DOCX 102 kb) [file 12903_2015_86_MOESM1_ESM.docx]

**Additional file 1: Table S1** All the differentially expressed genes (DEGs) in periodontitis samples with their corresponding modules

| **Gene symbol** | **log_2_ FC** | **AveExpr** | **t** | **P Value** | **FDR** | **Module** |
| --- | --- | --- | --- | --- | --- | --- |
| ARFGAP3 | 0.580009 | 8.528131 | 9.154782 | 7.39E-18 | 1.62E-16 |  |
| SOX18 | 0.580613 | 4.662644 | 5.828129 | 1.40E-08 | 8.27E-08 |  |
| HSP90B1 | 0.580918 | 9.433178 | 7.528222 | 5.58E-13 | 6.10E-12 |  |
| KIAA0040 | 0.581051 | 6.326406 | 13.26298 | 3.87E-32 | 9.81E-30 |  |
| SERINC2 | 0.581502 | 7.67743 | 7.321273 | 2.11E-12 | 2.13E-11 |  |
| HMHA1 | 0.582408 | 7.284838 | 9.36585 | 1.57E-18 | 3.78E-17 | 2 |
| ERO1LB | 0.582786 | 4.825818 | 8.585432 | 4.38E-16 | 7.42E-15 |  |
| NME4 | 0.583457 | 7.707356 | 9.415624 | 1.09E-18 | 2.67E-17 |  |
| FCGRT | 0.585794 | 7.663438 | 10.83633 | 1.96E-23 | 1.05E-21 |  |
| SELPLG | 0.585872 | 6.565889 | 11.11082 | 2.20E-24 | 1.41E-22 | 3 |
| SLAMF6 | 0.586385 | 6.482738 | 6.875586 | 3.38E-11 | 2.87E-10 |  |
| BTN3A3 | 0.588007 | 6.689441 | 5.969617 | 6.47E-09 | 4.02E-08 |  |
| WARS | 0.588225 | 8.376076 | 8.26645 | 4.03E-15 | 5.97E-14 |  |
| SELS | 0.589296 | 10.03914 | 8.05819 | 1.67E-14 | 2.27E-13 |  |
| CTSL1 | 0.589511 | 8.566502 | 10.17605 | 3.45E-21 | 1.23E-19 |  |
| DAP | 0.590168 | 8.468142 | 10.7317 | 4.50E-23 | 2.24E-21 |  |
| LASS2 | 0.590446 | 8.492475 | 12.8402 | 1.40E-30 | 2.58E-28 |  |
| GIMAP6 | 0.591392 | 7.203531 | 9.875147 | 3.45E-20 | 1.07E-18 |  |
| FCGR1B | 0.592688 | 5.853915 | 8.38044 | 1.83E-15 | 2.85E-14 |  |
| DUSP6 | 0.592783 | 8.157646 | 8.318221 | 2.82E-15 | 4.28E-14 |  |
| KCNJ8 | 0.592869 | 5.599324 | 11.64121 | 3.00E-26 | 2.74E-24 |  |
| TCF4 | 0.592906 | 8.443739 | 10.68212 | 6.65E-23 | 3.19E-21 |  |
| IFITM2 | 0.593002 | 10.66456 | 9.186842 | 5.85E-18 | 1.30E-16 |  |
| PIK3AP1 | 0.593253 | 5.735308 | 10.90089 | 1.18E-23 | 6.65E-22 |  |
| FAM171A1 | 0.593518 | 7.218778 | 10.6687 | 7.40E-23 | 3.52E-21 |  |
| HBD | 0.594449 | 5.270411 | 7.104583 | 8.24E-12 | 7.61E-11 |  |
| ESAM | 0.597509 | 6.797032 | 11.60967 | 3.88E-26 | 3.45E-24 |  |
| CSF1R | 0.598459 | 7.936744 | 9.421453 | 1.04E-18 | 2.56E-17 | 2 |
| IRF8 | 0.59871 | 7.703289 | 8.892781 | 4.93E-17 | 9.54E-16 | 2 |
| CYFIP2 | 0.59951 | 5.917306 | 11.07486 | 2.93E-24 | 1.86E-22 |  |
| CLEC1A | 0.600454 | 6.163918 | 8.563467 | 5.11E-16 | 8.56E-15 |  |
| IGHD | 0.600738 | 6.082594 | 10.52442 | 2.30E-22 | 1.00E-20 |  |
| CDC42EP3 | 0.600847 | 6.698004 | 12.11558 | 6.03E-28 | 7.75E-26 |  |
| CFH | 0.600934 | 8.314614 | 7.88234 | 5.43E-14 | 6.78E-13 |  |
| CHAC1 | 0.60134 | 6.942228 | 6.435002 | 4.65E-10 | 3.39E-09 |  |
| AIM2 | 0.601467 | 7.054483 | 7.336766 | 1.91E-12 | 1.94E-11 |  |
| SH3KBP1 | 0.601562 | 7.187804 | 11.83043 | 6.35E-27 | 6.48E-25 |  |
| LSP1 | 0.602838 | 7.537702 | 7.595314 | 3.61E-13 | 4.04E-12 |  |
| PPCDC | 0.603302 | 6.344084 | 7.926334 | 4.05E-14 | 5.16E-13 |  |
| FPR1 | 0.604038 | 5.857689 | 10.104 | 6.00E-21 | 2.10E-19 | 1 |
| SH2B3 | 0.604411 | 6.658125 | 10.87648 | 1.43E-23 | 7.99E-22 |  |
| LOC153684 | 0.604413 | 7.376033 | 9.954558 | 1.88E-20 | 6.04E-19 |  |
| LOC100132741 | 0.60463 | 4.672798 | 7.324412 | 2.06E-12 | 2.09E-11 |  |
| SEC24A | 0.604805 | 7.083914 | 8.61002 | 3.68E-16 | 6.33E-15 |  |
| EMCN | 0.604867 | 6.807046 | 9.74638 | 9.14E-20 | 2.65E-18 |  |
| GNE | 0.604976 | 6.711472 | 7.326706 | 2.03E-12 | 2.07E-11 |  |
| FOSB | 0.60532 | 8.094632 | 3.543128 | 0.000456 | 0.001273 |  |
| ASAM | 0.605478 | 5.992215 | 9.917852 | 2.49E-20 | 7.85E-19 |  |
| SULF1 | 0.605539 | 6.874034 | 8.916442 | 4.16E-17 | 8.15E-16 |  |
| MYO1F | 0.6061 | 6.912958 | 10.37679 | 7.28E-22 | 2.88E-20 | 3 |
| PPP1R16B | 0.606839 | 6.132375 | 10.99198 | 5.69E-24 | 3.39E-22 |  |
| C17orf62 | 0.607505 | 7.294525 | 9.872262 | 3.52E-20 | 1.09E-18 |  |
| CD53 | 0.607931 | 6.381742 | 13.38285 | 1.39E-32 | 3.81E-30 | 1 |
| LIME1 | 0.608652 | 6.868048 | 8.215609 | 5.71E-15 | 8.28E-14 |  |
| GIMAP4 | 0.609193 | 7.316518 | 10.03547 | 1.02E-20 | 3.40E-19 |  |
| SLC38A5 | 0.609496 | 7.40383 | 10.22837 | 2.30E-21 | 8.44E-20 |  |
| NCEH1 | 0.611967 | 4.593372 | 7.641956 | 2.66E-13 | 3.02E-12 |  |
| ECSCR | 0.61223 | 7.27788 | 12.6448 | 7.26E-30 | 1.20E-27 |  |
| LOC728264 | 0.612297 | 6.674741 | 12.03766 | 1.15E-27 | 1.36E-25 |  |
| CEACAM1 | 0.613522 | 7.95924 | 8.554188 | 5.45E-16 | 9.09E-15 |  |
| IL19 | 0.615047 | 6.226056 | 6.188103 | 1.91E-09 | 1.29E-08 |  |
| PLA2G7 | 0.615367 | 5.573869 | 7.234985 | 3.64E-12 | 3.56E-11 |  |
| THY1 | 0.616285 | 8.161538 | 10.9046 | 1.14E-23 | 6.48E-22 |  |
| NID1 | 0.616851 | 6.848724 | 14.21473 | 1.08E-35 | 7.82E-33 |  |
| PLEK | 0.619499 | 7.458604 | 10.40056 | 6.05E-22 | 2.42E-20 | 1 |
| GPR34 | 0.620067 | 6.259809 | 6.468475 | 3.83E-10 | 2.81E-09 |  |
| DENND3 | 0.620446 | 5.857094 | 12.87375 | 1.05E-30 | 1.98E-28 |  |
| SEC24D | 0.620681 | 5.575201 | 8.235664 | 4.97E-15 | 7.29E-14 |  |
| FAM117A | 0.621628 | 7.805095 | 11.25998 | 6.62E-25 | 4.68E-23 |  |
| WNT2 | 0.621792 | 5.288317 | 8.096785 | 1.28E-14 | 1.78E-13 |  |
| LILRB1 | 0.621839 | 6.454142 | 10.87471 | 1.45E-23 | 8.08E-22 |  |
| SOCS3 | 0.62265 | 6.384683 | 8.94446 | 3.40E-17 | 6.74E-16 |  |
| HSPG2 | 0.622653 | 7.524173 | 7.308619 | 2.28E-12 | 2.30E-11 |  |
| SPATS2 | 0.622709 | 6.120519 | 8.361986 | 2.08E-15 | 3.22E-14 |  |
| TNFRSF1B | 0.623684 | 6.961184 | 9.596009 | 2.83E-19 | 7.65E-18 |  |
| FAP | 0.624046 | 7.291501 | 8.295006 | 3.31E-15 | 4.96E-14 |  |
| FUCA2 | 0.624151 | 7.942239 | 10.31864 | 1.14E-21 | 4.41E-20 |  |
| WFDC1 | 0.624723 | 6.695998 | 10.00504 | 1.28E-20 | 4.22E-19 |  |
| PLD3 | 0.625963 | 7.880693 | 7.281899 | 2.70E-12 | 2.70E-11 |  |
| PREX1 | 0.626888 | 6.479332 | 11.957 | 2.24E-27 | 2.46E-25 |  |
| CCR7 | 0.626933 | 6.28847 | 6.215175 | 1.64E-09 | 1.12E-08 | 3 |
| LOC100287814 | 0.62803 | 5.499466 | 9.736589 | 9.84E-20 | 2.84E-18 |  |
| LOC96610 | 0.628372 | 6.857438 | 13.18778 | 7.34E-32 | 1.77E-29 |  |
| BMF | 0.62849 | 6.320536 | 10.51466 | 2.48E-22 | 1.08E-20 |  |
| DHRS9 | 0.628673 | 8.444727 | 8.086311 | 1.38E-14 | 1.90E-13 |  |
| KDELR1 | 0.628691 | 9.009054 | 9.468662 | 7.32E-19 | 1.85E-17 |  |
| ARID3A | 0.628716 | 5.682895 | 9.723232 | 1.09E-19 | 3.12E-18 |  |
| TMEM176B | 0.630849 | 6.660373 | 10.73826 | 4.27E-23 | 2.14E-21 |  |
| PAG1 | 0.631063 | 6.54161 | 8.647478 | 2.83E-16 | 4.93E-15 |  |
| SLC1A5 | 0.631153 | 7.573532 | 9.93986 | 2.11E-20 | 6.73E-19 |  |
| FKBP2 | 0.631402 | 8.616736 | 8.650659 | 2.76E-16 | 4.83E-15 |  |
| HAS2 | 0.631412 | 6.203686 | 6.322821 | 8.89E-10 | 6.24E-09 |  |
| GNG7 | 0.632625 | 5.845881 | 10.21455 | 2.56E-21 | 9.34E-20 |  |
| SFMBT2 | 0.63367 | 5.318846 | 11.13599 | 1.80E-24 | 1.17E-22 |  |
| HSD17B11 | 0.633805 | 7.429188 | 9.527615 | 4.72E-19 | 1.23E-17 |  |
| FAM20A | 0.634059 | 6.811364 | 9.598689 | 2.77E-19 | 7.51E-18 |  |
| TMEM205 | 0.635 | 8.617703 | 9.526095 | 4.77E-19 | 1.24E-17 |  |
| ARSA | 0.635358 | 6.797806 | 8.939465 | 3.52E-17 | 6.97E-16 |  |
| RAI14 | 0.637694 | 8.142208 | 10.38566 | 6.79E-22 | 2.69E-20 |  |
| TRAM2 | 0.638289 | 7.234924 | 8.913715 | 4.24E-17 | 8.31E-16 |  |
| CCL5 | 0.638899 | 6.897292 | 7.266216 | 2.99E-12 | 2.96E-11 | 1 |
| C1QB | 0.639285 | 6.970542 | 6.985532 | 1.72E-11 | 1.52E-10 | 2 |
| BASP1 | 0.639383 | 9.783131 | 11.20982 | 9.92E-25 | 6.83E-23 |  |
| LRMP | 0.641401 | 5.39561 | 10.72106 | 4.89E-23 | 2.42E-21 |  |
| CLIC4 | 0.642026 | 8.184817 | 9.432614 | 9.57E-19 | 2.37E-17 |  |
| ARHGDIB | 0.642515 | 8.744589 | 13.67172 | 1.17E-33 | 4.57E-31 | 2 |
| SHC1 | 0.644565 | 8.843985 | 10.40745 | 5.73E-22 | 2.31E-20 |  |
| C11orf96 | 0.644587 | 7.366654 | 5.904494 | 9.24E-09 | 5.61E-08 |  |
| MMP14 | 0.644962 | 7.378085 | 10.7111 | 5.29E-23 | 2.60E-21 |  |
| HLA-DRA | 0.645034 | 11.05137 | 6.152779 | 2.34E-09 | 1.55E-08 |  |
| PTPRCAP | 0.645776 | 6.90626 | 9.525796 | 4.78E-19 | 1.25E-17 |  |
| IL32 | 0.646121 | 6.981396 | 8.158661 | 8.42E-15 | 1.19E-13 |  |
| ENG | 0.647201 | 7.184342 | 9.033734 | 1.78E-17 | 3.68E-16 |  |
| PTGS2 | 0.648192 | 5.454629 | 4.853145 | 1.92E-06 | 8.07E-06 | 3 |
| ALOX5AP | 0.650455 | 7.724843 | 9.482345 | 6.61E-19 | 1.68E-17 | 2 |
| STK17B | 0.650927 | 5.760167 | 10.33869 | 9.79E-22 | 3.82E-20 |  |
| QPRT | 0.651302 | 5.978287 | 11.16267 | 1.45E-24 | 9.68E-23 |  |
| FERMT3 | 0.651718 | 6.622676 | 11.2226 | 8.95E-25 | 6.18E-23 | 2 |
| SHANK3 | 0.652038 | 7.241664 | 10.30777 | 1.24E-21 | 4.75E-20 |  |
| WIPF1 | 0.652039 | 6.295657 | 12.61478 | 9.34E-30 | 1.49E-27 |  |
| KRT19 | 0.653415 | 7.321857 | 9.688993 | 1.41E-19 | 3.95E-18 |  |
| ARMCX3 | 0.654817 | 7.352741 | 8.860293 | 6.22E-17 | 1.19E-15 |  |
| GUCY1B3 | 0.656333 | 6.245917 | 11.31741 | 4.16E-25 | 3.11E-23 |  |
| FLJ32255 | 0.657507 | 5.220881 | 9.527945 | 4.71E-19 | 1.23E-17 |  |
| MIR155HG | 0.657971 | 4.493147 | 6.507222 | 3.05E-10 | 2.28E-09 |  |
| GPX7 | 0.658047 | 5.980384 | 7.721682 | 1.58E-13 | 1.85E-12 |  |
| FAIM3 | 0.659672 | 6.941918 | 9.499519 | 5.82E-19 | 1.50E-17 |  |
| ASS1 | 0.659945 | 9.791711 | 7.577708 | 4.05E-13 | 4.50E-12 |  |
| MOGS | 0.660513 | 7.353533 | 9.527929 | 4.71E-19 | 1.23E-17 |  |
| PDGFD | 0.661437 | 6.570698 | 11.2297 | 8.45E-25 | 5.86E-23 |  |
| MGP | 0.662444 | 6.474968 | 10.27434 | 1.61E-21 | 6.02E-20 |  |
| RGS5 | 0.663754 | 8.220338 | 10.34938 | 9.01E-22 | 3.53E-20 |  |
| SMPDL3B | 0.666422 | 6.255418 | 10.13987 | 4.55E-21 | 1.61E-19 |  |
| TMEM140 | 0.666448 | 6.206066 | 11.36065 | 2.94E-25 | 2.24E-23 |  |
| KCNA3 | 0.666509 | 4.994279 | 8.30547 | 3.08E-15 | 4.64E-14 |  |
| RPN2 | 0.666596 | 10.84257 | 10.42101 | 5.16E-22 | 2.10E-20 |  |
| TSPAN11 | 0.667481 | 7.12393 | 11.39187 | 2.28E-25 | 1.78E-23 |  |
| ST8SIA4 | 0.668029 | 4.601827 | 9.828397 | 4.92E-20 | 1.49E-18 |  |
| GLRX | 0.669259 | 8.82061 | 8.091196 | 1.33E-14 | 1.84E-13 |  |
| RPN1 | 0.671083 | 9.361155 | 8.572728 | 4.79E-16 | 8.06E-15 |  |
| TSPAN13 | 0.673481 | 9.249941 | 11.39533 | 2.22E-25 | 1.74E-23 |  |
| EDEM2 | 0.674508 | 6.918128 | 9.661054 | 1.74E-19 | 4.80E-18 |  |
| CMTM7 | 0.674909 | 7.082413 | 10.07784 | 7.34E-21 | 2.52E-19 |  |
| LDB2 | 0.67565 | 7.338142 | 10.33848 | 9.80E-22 | 3.82E-20 |  |
| FTL | 0.677004 | 11.98493 | 11.16205 | 1.46E-24 | 9.70E-23 |  |
| F13A1 | 0.677134 | 8.780341 | 10.30765 | 1.25E-21 | 4.75E-20 |  |
| FBN1 | 0.679934 | 7.793615 | 10.48607 | 3.10E-22 | 1.32E-20 |  |
| CCDC69 | 0.680581 | 5.83015 | 11.40944 | 1.98E-25 | 1.56E-23 |  |
| C4orf7 | 0.680926 | 13.39652 | 10.87309 | 1.47E-23 | 8.16E-22 |  |
| PKIG | 0.681193 | 8.068184 | 12.07494 | 8.45E-28 | 1.02E-25 |  |
| PDIA4 | 0.681354 | 8.025272 | 10.88907 | 1.29E-23 | 7.27E-22 |  |
| GANAB | 0.681393 | 9.063701 | 11.03765 | 3.95E-24 | 2.45E-22 |  |
| SLC7A11 | 0.681953 | 7.580139 | 9.337837 | 1.93E-18 | 4.61E-17 |  |
| TXNIP | 0.682145 | 10.40717 | 8.194948 | 6.57E-15 | 9.46E-14 |  |
| ADAMTS1 | 0.682996 | 6.7735 | 7.476622 | 7.79E-13 | 8.39E-12 |  |
| MAST1 | 0.685005 | 6.843022 | 9.497803 | 5.89E-19 | 1.51E-17 |  |
| CHST11 | 0.68591 | 6.782293 | 11.77494 | 1.00E-26 | 9.93E-25 |  |
| ITGA4 | 0.686516 | 5.014777 | 8.744531 | 1.42E-16 | 2.57E-15 | 2 |
| ALDH1A3 | 0.688393 | 8.496532 | 7.963957 | 3.15E-14 | 4.05E-13 |  |
| GPR116 | 0.688513 | 6.988551 | 12.65086 | 6.90E-30 | 1.15E-27 |  |
| ZNF215 | 0.688978 | 4.960988 | 10.69007 | 6.25E-23 | 3.02E-21 |  |
| BTN3A2 | 0.689051 | 7.576059 | 7.889781 | 5.17E-14 | 6.48E-13 |  |
| RGS18 | 0.68935 | 5.158311 | 7.807244 | 8.96E-14 | 1.09E-12 | 1 |
| SERPINH1 | 0.692928 | 8.666553 | 8.305222 | 3.08E-15 | 4.64E-14 |  |
| GPSM3 | 0.692948 | 7.170157 | 11.28172 | 5.56E-25 | 3.99E-23 |  |
| MNDA | 0.694198 | 7.309419 | 6.761916 | 6.72E-11 | 5.48E-10 | 2 |
| LCK | 0.694243 | 6.310563 | 10.30043 | 1.32E-21 | 5.01E-20 | 2 |
| TDO2 | 0.694289 | 4.331942 | 11.41381 | 1.91E-25 | 1.52E-23 |  |
| TCIRG1 | 0.69499 | 7.543848 | 9.625539 | 2.27E-19 | 6.21E-18 |  |
| MS4A4A | 0.69502 | 6.382622 | 8.961414 | 3.01E-17 | 6.03E-16 |  |
| GVINP1 | 0.69719 | 6.237472 | 10.02856 | 1.07E-20 | 3.57E-19 |  |
| IGLV4-3 | 0.698539 | 7.060227 | 9.577054 | 3.26E-19 | 8.75E-18 |  |
| WFS1 | 0.698667 | 6.538656 | 10.77489 | 3.20E-23 | 1.64E-21 |  |
| FAM198B | 0.699011 | 6.840935 | 10.02066 | 1.14E-20 | 3.76E-19 |  |
| ADAMDEC1 | 0.699844 | 6.90688 | 6.171226 | 2.11E-09 | 1.41E-08 |  |
| DOK3 | 0.70039 | 6.168462 | 10.59087 | 1.36E-22 | 6.19E-21 |  |
| CXCL3 | 0.700525 | 6.250318 | 5.557337 | 5.88E-08 | 3.15E-07 | 1 |
| SIDT1 | 0.70146 | 4.949396 | 9.060701 | 1.47E-17 | 3.07E-16 |  |
| OGN | 0.701583 | 7.013355 | 4.863171 | 1.84E-06 | 7.73E-06 |  |
| PIP5K1B | 0.702525 | 4.462294 | 10.5482 | 1.91E-22 | 8.50E-21 |  |
| MPPED2 | 0.705546 | 4.671145 | 8.881546 | 5.34E-17 | 1.03E-15 |  |
| AQP9 | 0.711256 | 6.375833 | 7.706605 | 1.74E-13 | 2.03E-12 |  |
| ECM1 | 0.711975 | 10.68361 | 7.306137 | 2.32E-12 | 2.34E-11 |  |
| FXYD5 | 0.712596 | 8.643434 | 8.821588 | 8.20E-17 | 1.55E-15 |  |
| NID2 | 0.71309 | 6.964135 | 9.497741 | 5.90E-19 | 1.51E-17 |  |
| CTHRC1 | 0.713843 | 9.060626 | 7.991656 | 2.61E-14 | 3.43E-13 |  |
| RAMP3 | 0.713939 | 7.662981 | 12.55856 | 1.50E-29 | 2.38E-27 |  |
| ERLEC1 | 0.71427 | 7.538783 | 12.21854 | 2.57E-28 | 3.43E-26 |  |
| MLKL | 0.714346 | 8.103657 | 11.93269 | 2.74E-27 | 2.88E-25 |  |
| C8orf4 | 0.714809 | 6.470328 | 7.584938 | 3.86E-13 | 4.30E-12 |  |
| GAS6 | 0.71703 | 8.769654 | 10.70572 | 5.52E-23 | 2.70E-21 |  |
| TMEM156 | 0.717267 | 4.706286 | 8.759147 | 1.28E-16 | 2.33E-15 |  |
| CHI3L2 | 0.717349 | 6.236255 | 8.034948 | 1.95E-14 | 2.63E-13 |  |
| FAM55C | 0.718586 | 5.086958 | 9.855675 | 4.00E-20 | 1.22E-18 |  |
| ATP2A3 | 0.719402 | 6.027449 | 10.42414 | 5.03E-22 | 2.05E-20 |  |
| TAGLN | 0.71987 | 7.845193 | 10.48578 | 3.11E-22 | 1.32E-20 |  |
| RNF166 | 0.719934 | 5.872591 | 8.919642 | 4.06E-17 | 7.97E-16 |  |
| CD2 | 0.720417 | 7.254685 | 8.876483 | 5.54E-17 | 1.07E-15 | 3 |
| TPST2 | 0.720944 | 8.514006 | 10.98519 | 6.01E-24 | 3.55E-22 |  |
| C9orf150 | 0.722138 | 6.531582 | 11.26536 | 6.34E-25 | 4.50E-23 |  |
| CD97 | 0.723377 | 7.168932 | 10.0275 | 1.08E-20 | 3.60E-19 |  |
| SFRP2 | 0.724284 | 7.967958 | 7.577514 | 4.05E-13 | 4.50E-12 |  |
| C10orf10 | 0.72514 | 7.15893 | 12.3333 | 9.86E-29 | 1.41E-26 |  |
| CHN1 | 0.725216 | 8.257849 | 11.62187 | 3.51E-26 | 3.17E-24 |  |
| GIMAP2 | 0.725569 | 6.432746 | 7.877951 | 5.60E-14 | 6.98E-13 |  |
| CTH | 0.72624 | 4.395691 | 8.399823 | 1.60E-15 | 2.52E-14 |  |
| TBC1D10C | 0.728178 | 6.812352 | 10.92238 | 9.91E-24 | 5.67E-22 |  |
| BTK | 0.728605 | 6.000647 | 10.07525 | 7.48E-21 | 2.57E-19 |  |
| CCR1 | 0.728616 | 6.221008 | 9.992145 | 1.41E-20 | 4.62E-19 | 1 |
| PLA1A | 0.730497 | 5.101392 | 9.436845 | 9.27E-19 | 2.30E-17 |  |
| EGR1 | 0.730554 | 9.367574 | 7.393838 | 1.33E-12 | 1.38E-11 |  |
| MIR21 | 0.731339 | 10.29154 | 8.539931 | 6.03E-16 | 1.00E-14 |  |
| VCAM1 | 0.733176 | 7.710988 | 8.438159 | 1.23E-15 | 1.97E-14 | 2 |
| SAMSN1 | 0.733592 | 5.642241 | 9.97187 | 1.65E-20 | 5.36E-19 |  |
| LBH | 0.733918 | 6.693954 | 12.68672 | 5.10E-30 | 8.78E-28 |  |
| MAN1A1 | 0.736341 | 7.674125 | 9.323182 | 2.15E-18 | 5.12E-17 |  |
| EDEM1 | 0.736744 | 7.808026 | 7.686724 | 1.98E-13 | 2.30E-12 |  |
| ADA | 0.7393 | 6.658471 | 12.87659 | 1.03E-30 | 1.95E-28 |  |
| GPR65 | 0.739321 | 5.772974 | 9.873104 | 3.50E-20 | 1.09E-18 | 2 |
| BANK1 | 0.739403 | 5.855706 | 9.574375 | 3.33E-19 | 8.92E-18 |  |
| C12orf23 | 0.739802 | 7.97433 | 9.022587 | 1.93E-17 | 3.97E-16 |  |
| PDE4B | 0.740883 | 5.968572 | 8.911796 | 4.30E-17 | 8.41E-16 |  |
| ELTD1 | 0.742141 | 7.871503 | 10.51828 | 2.41E-22 | 1.05E-20 |  |
| KLRB1 | 0.74275 | 6.002943 | 8.760413 | 1.27E-16 | 2.32E-15 | 3 |
| EMP3 | 0.745 | 8.570077 | 7.529265 | 5.54E-13 | 6.06E-12 |  |
| TXNDC15 | 0.74584 | 8.491873 | 10.57389 | 1.56E-22 | 7.02E-21 |  |
| BCL2A1 | 0.745865 | 5.297379 | 6.36399 | 7.02E-10 | 4.99E-09 |  |
| IFNAR2 | 0.747426 | 7.310934 | 11.59694 | 4.30E-26 | 3.80E-24 |  |
| VNN1 | 0.750144 | 6.741417 | 7.97967 | 2.83E-14 | 3.70E-13 |  |
| SLC2A3 | 0.750677 | 6.449646 | 9.665177 | 1.69E-19 | 4.67E-18 |  |
| RAB30 | 0.750782 | 5.997583 | 11.33721 | 3.55E-25 | 2.69E-23 |  |
| MARCKSL1 | 0.751534 | 7.961604 | 11.52141 | 7.96E-26 | 6.82E-24 |  |
| LBP | 0.751733 | 6.281247 | 10.17859 | 3.38E-21 | 1.21E-19 |  |
| CD48 | 0.752198 | 6.555099 | 12.06238 | 9.37E-28 | 1.13E-25 | 1 |
| PDGFRB | 0.752453 | 8.329271 | 12.75008 | 2.99E-30 | 5.28E-28 |  |
| UGT2B17 | 0.75275 | 3.970931 | 6.520828 | 2.82E-10 | 2.11E-09 |  |
| RABAC1 | 0.75308 | 9.645437 | 7.698209 | 1.84E-13 | 2.14E-12 |  |
| SLA | 0.754464 | 7.291975 | 11.94745 | 2.42E-27 | 2.62E-25 | 3 |
| LGALS1 | 0.757629 | 10.83797 | 8.034585 | 1.96E-14 | 2.63E-13 |  |
| KISS1R | 0.757834 | 5.012934 | 7.93991 | 3.70E-14 | 4.72E-13 |  |
| CYP4X1 | 0.757856 | 7.111339 | 8.159506 | 8.37E-15 | 1.19E-13 |  |
| FMOD | 0.758985 | 9.454905 | 13.38659 | 1.35E-32 | 3.74E-30 |  |
| IGDCC4 | 0.761137 | 5.765847 | 7.348007 | 1.78E-12 | 1.82E-11 |  |
| HLA-DOB | 0.762892 | 6.335377 | 12.73162 | 3.50E-30 | 6.07E-28 |  |
| GZMB | 0.763921 | 7.10222 | 8.021005 | 2.14E-14 | 2.86E-13 | 2 |
| SSR3 | 0.764374 | 8.027224 | 10.49036 | 3.00E-22 | 1.28E-20 |  |
| PDIA5 | 0.765441 | 7.629355 | 11.42691 | 1.72E-25 | 1.37E-23 |  |
| MEI1 | 0.765782 | 6.271914 | 10.81844 | 2.26E-23 | 1.19E-21 |  |
| CSF3R | 0.768732 | 6.632618 | 9.944575 | 2.03E-20 | 6.50E-19 |  |
| NCF2 | 0.770679 | 6.382704 | 9.695643 | 1.34E-19 | 3.78E-18 | 1 |
| NCKAP1L | 0.770714 | 7.075605 | 11.30387 | 4.65E-25 | 3.42E-23 | 1 |
| LCP1 | 0.771322 | 8.615462 | 11.3067 | 4.54E-25 | 3.35E-23 | 1 |
| CDH5 | 0.771329 | 6.811142 | 11.29203 | 5.11E-25 | 3.69E-23 |  |
| CRELD2 | 0.772202 | 9.435829 | 9.743896 | 9.32E-20 | 2.69E-18 |  |
| LOC100286909 | 0.773488 | 7.60495 | 9.845397 | 4.32E-20 | 1.32E-18 |  |
| SLC15A2 | 0.774102 | 5.331529 | 9.349309 | 1.77E-18 | 4.25E-17 |  |
| SEPT6 | 0.774214 | 5.942147 | 11.77727 | 9.83E-27 | 9.79E-25 |  |
| NCF1C | 0.776146 | 6.000626 | 9.379675 | 1.42E-18 | 3.43E-17 |  |
| CD52 | 0.778075 | 7.563699 | 8.575027 | 4.71E-16 | 7.96E-15 | 1 |
| TAPBPL | 0.779743 | 7.121513 | 9.799032 | 6.14E-20 | 1.83E-18 |  |
| GMFG | 0.781512 | 7.528687 | 11.77808 | 9.77E-27 | 9.77E-25 | 1 |
| GZMK | 0.78196 | 6.752236 | 7.966582 | 3.09E-14 | 3.99E-13 |  |
| VIM | 0.78646 | 9.247542 | 10.12733 | 5.02E-21 | 1.77E-19 |  |
| C4orf48 | 0.787315 | 7.236771 | 10.82199 | 2.20E-23 | 1.17E-21 |  |
| FMO1 | 0.787992 | 7.031002 | 7.545184 | 5.00E-13 | 5.50E-12 |  |
| IL7R | 0.788696 | 7.753989 | 8.261875 | 4.15E-15 | 6.13E-14 | 2 |
| CCPG1 | 0.789205 | 7.133881 | 9.162834 | 6.97E-18 | 1.53E-16 |  |
| TIMP1 | 0.791678 | 10.35319 | 8.349119 | 2.28E-15 | 3.50E-14 | 3 |
| AKR1B1 | 0.791865 | 8.77838 | 12.79086 | 2.12E-30 | 3.85E-28 |  |
| FCER1G | 0.792711 | 7.191899 | 10.5624 | 1.71E-22 | 7.65E-21 | 2 |
| ENTPD1 | 0.793126 | 7.013193 | 14.01466 | 6.09E-35 | 3.53E-32 |  |
| EVI2A | 0.80025 | 7.104653 | 8.711091 | 1.80E-16 | 3.22E-15 |  |
| SPCS3 | 0.802081 | 8.450184 | 10.40663 | 5.77E-22 | 2.32E-20 |  |
| E2F5 | 0.802273 | 6.133675 | 8.988855 | 2.47E-17 | 5.00E-16 |  |
| NEU1 | 0.803197 | 8.015281 | 10.17278 | 3.53E-21 | 1.26E-19 |  |
| C4orf26 | 0.803407 | 8.56674 | 5.021663 | 8.64E-07 | 3.84E-06 |  |
| HLA-DMA | 0.804617 | 8.614177 | 10.31407 | 1.18E-21 | 4.55E-20 |  |
| LAPTM5 | 0.80518 | 9.073761 | 11.05414 | 3.46E-24 | 2.15E-22 | 1 |
| MPEG1 | 0.805793 | 7.719516 | 11.10733 | 2.26E-24 | 1.44E-22 | 1 |
| HYOU1 | 0.806095 | 9.351315 | 10.27482 | 1.61E-21 | 6.02E-20 |  |
| C1S | 0.806523 | 9.790298 | 9.099399 | 1.11E-17 | 2.35E-16 |  |
| IL24 | 0.807893 | 5.674431 | 8.045163 | 1.82E-14 | 2.46E-13 |  |
| P2RX4 | 0.809749 | 7.141105 | 11.99601 | 1.62E-27 | 1.83E-25 |  |
| SMAP2 | 0.809906 | 8.010641 | 9.914577 | 2.56E-20 | 8.03E-19 |  |
| ENO2 | 0.810908 | 5.490577 | 9.264705 | 3.30E-18 | 7.62E-17 |  |
| PLVAP | 0.811396 | 8.096678 | 10.05714 | 8.60E-21 | 2.92E-19 |  |
| IL6 | 0.813015 | 6.745565 | 6.122631 | 2.77E-09 | 1.82E-08 | 2 |
| HCK | 0.820912 | 6.848077 | 12.24441 | 2.07E-28 | 2.82E-26 | 2 |
| C1QC | 0.821275 | 7.723851 | 9.116068 | 9.80E-18 | 2.11E-16 | 3 |
| IFI30 | 0.822981 | 9.25861 | 11.76895 | 1.05E-26 | 1.03E-24 | 2 |
| NNMT | 0.82397 | 8.530613 | 9.294768 | 2.65E-18 | 6.22E-17 |  |
| LOC100506098 | 0.825289 | 8.234646 | 8.349873 | 2.26E-15 | 3.49E-14 |  |
| PKHD1L1 | 0.827872 | 5.091325 | 6.983599 | 1.74E-11 | 1.54E-10 |  |
| RASSF2 | 0.828124 | 7.653998 | 12.65382 | 6.73E-30 | 1.13E-27 |  |
| FAM107B | 0.82844 | 7.818957 | 10.09321 | 6.52E-21 | 2.26E-19 |  |
| IRF4 | 0.828527 | 5.598606 | 11.23183 | 8.31E-25 | 5.80E-23 |  |
| C5AR1 | 0.828817 | 6.414717 | 9.956848 | 1.85E-20 | 5.96E-19 | 1 |
| BEX5 | 0.829504 | 6.274286 | 4.846785 | 1.98E-06 | 8.30E-06 |  |
| SLC2A10 | 0.832216 | 7.048632 | 10.60974 | 1.18E-22 | 5.44E-21 |  |
| EPDR1 | 0.833301 | 6.795197 | 10.22164 | 2.42E-21 | 8.85E-20 |  |
| HBB | 0.838893 | 10.5807 | 7.929889 | 3.95E-14 | 5.04E-13 |  |
| CHST15 | 0.8399 | 9.091027 | 14.3092 | 4.75E-36 | 4.02E-33 |  |
| SLFN11 | 0.841229 | 6.847758 | 10.76965 | 3.33E-23 | 1.70E-21 |  |
| FABP4 | 0.841294 | 5.503804 | 8.560684 | 5.21E-16 | 8.72E-15 |  |
| ARHGAP15 | 0.842919 | 6.841412 | 11.43659 | 1.59E-25 | 1.28E-23 |  |
| G0S2 | 0.84937 | 7.745712 | 8.489138 | 8.60E-16 | 1.41E-14 |  |
| VNN2 | 0.850362 | 5.683496 | 10.66607 | 7.55E-23 | 3.58E-21 |  |
| FICD | 0.851685 | 7.237551 | 8.764105 | 1.24E-16 | 2.26E-15 |  |
| UCP2 | 0.853042 | 7.886734 | 10.07111 | 7.73E-21 | 2.64E-19 |  |
| KDR | 0.855625 | 7.542581 | 14.25861 | 7.37E-36 | 5.99E-33 |  |
| SELP | 0.85777 | 8.066446 | 13.83048 | 2.98E-34 | 1.38E-31 | 3 |
| MICB | 0.860527 | 5.991071 | 10.00426 | 1.29E-20 | 4.23E-19 |  |
| COTL1 | 0.863107 | 7.846769 | 10.82288 | 2.19E-23 | 1.16E-21 |  |
| PDK1 | 0.865084 | 7.882808 | 9.398906 | 1.23E-18 | 2.99E-17 |  |
| GYPC | 0.865582 | 7.663106 | 11.93995 | 2.58E-27 | 2.76E-25 |  |
| GLCCI1 | 0.867758 | 6.60813 | 10.78198 | 3.02E-23 | 1.57E-21 |  |
| C1QA | 0.869641 | 7.545041 | 10.12311 | 5.18E-21 | 1.82E-19 | 3 |
| XBP1 | 0.871549 | 8.182786 | 13.15411 | 9.77E-32 | 2.23E-29 |  |
| TNFAIP6 | 0.873094 | 7.364052 | 8.514341 | 7.21E-16 | 1.19E-14 |  |
| SLC7A7 | 0.878897 | 7.682509 | 11.50997 | 8.74E-26 | 7.46E-24 | 2 |
| FNDC3B | 0.881798 | 7.181305 | 13.67658 | 1.12E-33 | 4.56E-31 |  |
| C19orf10 | 0.8858 | 8.953887 | 10.93402 | 9.03E-24 | 5.20E-22 |  |
| LY9 | 0.885955 | 5.884872 | 11.0633 | 3.22E-24 | 2.02E-22 |  |
| PARM1 | 0.8879 | 6.641272 | 10.49063 | 2.99E-22 | 1.28E-20 |  |
| CD93 | 0.88794 | 8.406824 | 15.79457 | 1.08E-41 | 1.09E-37 |  |
| CD180 | 0.888384 | 6.077852 | 10.16716 | 3.69E-21 | 1.31E-19 |  |
| FAM30A | 0.889811 | 7.317498 | 9.59018 | 2.96E-19 | 7.97E-18 |  |
| FAM113B | 0.891365 | 6.249841 | 10.35307 | 8.75E-22 | 3.44E-20 |  |
| ITGAL | 0.893881 | 6.002783 | 12.39256 | 6.01E-29 | 8.92E-27 |  |
| KLHL6 | 0.89643 | 5.501522 | 10.97251 | 6.65E-24 | 3.90E-22 |  |
| DARC | 0.897185 | 8.511804 | 12.93504 | 6.27E-31 | 1.27E-28 |  |
| MEF2C | 0.897346 | 7.07331 | 13.63975 | 1.54E-33 | 5.68E-31 |  |
| ELMO1 | 0.897609 | 5.944832 | 13.09002 | 1.68E-31 | 3.68E-29 |  |
| CD200 | 0.898609 | 6.478847 | 13.50191 | 5.02E-33 | 1.57E-30 |  |
| XDH | 0.902374 | 7.759546 | 10.94945 | 7.99E-24 | 4.63E-22 |  |
| ENPEP | 0.902593 | 5.816434 | 8.761048 | 1.26E-16 | 2.31E-15 |  |
| GLDC | 0.903752 | 6.009667 | 10.80531 | 2.51E-23 | 1.31E-21 |  |
| TRBC1 | 0.904505 | 8.19836 | 10.5109 | 2.56E-22 | 1.10E-20 |  |
| CYBA | 0.906432 | 8.195696 | 12.03684 | 1.16E-27 | 1.36E-25 |  |
| MS4A1 | 0.906852 | 6.099347 | 7.958019 | 3.27E-14 | 4.21E-13 |  |
| HSH2D | 0.907411 | 6.259709 | 10.46319 | 3.71E-22 | 1.55E-20 |  |
| CCL19 | 0.908081 | 8.372579 | 7.821832 | 8.13E-14 | 9.90E-13 |  |
| RASSF6 | 0.910094 | 5.866295 | 8.466222 | 1.01E-15 | 1.64E-14 |  |
| FCRLB | 0.911061 | 6.257329 | 9.589276 | 2.98E-19 | 8.02E-18 |  |
| ISG20 | 0.911822 | 8.518034 | 8.785071 | 1.06E-16 | 1.97E-15 |  |
| SLC12A8 | 0.914366 | 7.321538 | 9.237882 | 4.02E-18 | 9.08E-17 |  |
| PCDH17 | 0.916142 | 6.802184 | 12.08771 | 7.60E-28 | 9.30E-26 |  |
| DDIT4 | 0.916816 | 9.802927 | 10.12466 | 5.12E-21 | 1.80E-19 |  |
| CTSS | 0.922067 | 7.772236 | 12.02685 | 1.26E-27 | 1.46E-25 | 1 |
| PTGDS | 0.922403 | 8.776886 | 9.239882 | 3.96E-18 | 8.97E-17 |  |
| TMEM176A | 0.923627 | 8.366519 | 11.66344 | 2.50E-26 | 2.31E-24 |  |
| ADAM28 | 0.926207 | 6.212921 | 13.26756 | 3.72E-32 | 9.56E-30 |  |
| CD69 | 0.927037 | 5.787336 | 6.833194 | 4.37E-11 | 3.66E-10 | 2 |
| MMP9 | 0.928905 | 7.867404 | 9.02958 | 1.84E-17 | 3.79E-16 | 3 |
| SDF2L1 | 0.928968 | 8.032099 | 9.274534 | 3.07E-18 | 7.12E-17 |  |
| BIN2 | 0.93464 | 6.927176 | 13.2205 | 5.55E-32 | 1.38E-29 | 2 |
| IGLV6-57 | 0.935897 | 7.964612 | 10.50578 | 2.66E-22 | 1.15E-20 |  |
| RGS2 | 0.936026 | 9.430122 | 8.775342 | 1.14E-16 | 2.10E-15 |  |
| RARRES2 | 0.938736 | 7.927427 | 12.63451 | 7.92E-30 | 1.30E-27 |  |
| CLCA4 | 0.942758 | 10.39562 | 8.084459 | 1.39E-14 | 1.92E-13 |  |
| HSD11B1 | 0.944232 | 6.218719 | 12.36984 | 7.27E-29 | 1.06E-26 |  |
| LYN | 0.949778 | 7.665337 | 14.88987 | 3.02E-38 | 7.68E-35 |  |
| MYL9 | 0.950588 | 7.564089 | 9.409341 | 1.14E-18 | 2.78E-17 |  |
| ARHGAP9 | 0.952449 | 6.653195 | 12.43746 | 4.13E-29 | 6.26E-27 | 2 |
| PXDN | 0.959971 | 7.669612 | 12.01331 | 1.41E-27 | 1.61E-25 |  |
| TXNDC11 | 0.962116 | 8.353115 | 11.23127 | 8.35E-25 | 5.80E-23 |  |
| APLNR | 0.96394 | 7.304189 | 12.9989 | 3.65E-31 | 7.72E-29 |  |
| OLFML2B | 0.966191 | 7.731665 | 11.67401 | 2.29E-26 | 2.13E-24 |  |
| FNDC1 | 0.967373 | 5.898601 | 8.586075 | 4.36E-16 | 7.39E-15 |  |
| ALOX15B | 0.970609 | 7.253447 | 9.46865 | 7.32E-19 | 1.85E-17 |  |
| AMPD1 | 0.97132 | 5.904677 | 9.102507 | 1.08E-17 | 2.30E-16 |  |
| CD37 | 0.976745 | 6.22319 | 10.255 | 1.87E-21 | 6.92E-20 | 1 |
| SEL1L | 0.983928 | 7.324279 | 9.636321 | 2.09E-19 | 5.74E-18 |  |
| VOPP1 | 0.984438 | 9.253691 | 12.27328 | 1.63E-28 | 2.23E-26 |  |
| CD14 | 0.987422 | 8.365159 | 10.86637 | 1.55E-23 | 8.49E-22 | 2 |
| STAP1 | 0.988416 | 5.651878 | 10.71154 | 5.28E-23 | 2.60E-21 |  |
| P2RX5 | 0.990644 | 6.775655 | 10.59892 | 1.28E-22 | 5.87E-21 |  |
| FBXO16 | 0.990708 | 4.891348 | 10.75983 | 3.60E-23 | 1.83E-21 |  |
| PRDX4 | 0.996651 | 10.64369 | 11.40021 | 2.13E-25 | 1.68E-23 |  |
| MGC16075 | 0.999394 | 4.672222 | 7.112128 | 7.86E-12 | 7.27E-11 |  |
| SSR4 | 1.00667 | 11.73151 | 11.27835 | 5.71E-25 | 4.07E-23 |  |
| SASH3 | 1.018271 | 7.065231 | 12.44058 | 4.03E-29 | 6.15E-27 | 2 |
| FUT8 | 1.025256 | 6.90518 | 11.93671 | 2.65E-27 | 2.82E-25 |  |
| PROK2 | 1.032425 | 4.595911 | 7.684949 | 2.01E-13 | 2.32E-12 |  |
| GPR160 | 1.033578 | 6.455162 | 9.690202 | 1.40E-19 | 3.92E-18 |  |
| DERL3 | 1.04121 | 6.773051 | 11.93449 | 2.70E-27 | 2.85E-25 |  |
| IGFBP4 | 1.04189 | 9.593901 | 9.070689 | 1.36E-17 | 2.86E-16 |  |
| DDIT4L | 1.045486 | 7.571163 | 10.4311 | 4.77E-22 | 1.96E-20 |  |
| ZBP1 | 1.048171 | 5.73252 | 10.41281 | 5.50E-22 | 2.22E-20 |  |
| PPBP | 1.050761 | 5.189827 | 7.706699 | 1.74E-13 | 2.03E-12 | 1 |
| FCN1 | 1.052891 | 6.131444 | 12.32628 | 1.05E-28 | 1.47E-26 | 2 |
| IL1B | 1.054361 | 8.834313 | 9.702617 | 1.27E-19 | 3.60E-18 | 3 |
| CTSH | 1.056088 | 9.649514 | 15.67822 | 3.00E-41 | 2.03E-37 |  |
| CFI | 1.056224 | 7.177591 | 11.20118 | 1.06E-24 | 7.30E-23 |  |
| CTGF | 1.070551 | 8.31592 | 9.871445 | 3.55E-20 | 1.10E-18 |  |
| SRGN | 1.077947 | 8.355648 | 13.92652 | 1.30E-34 | 6.79E-32 | 2 |
| CORO1A | 1.078724 | 7.706375 | 11.8056 | 7.79E-27 | 7.83E-25 | 2 |
| ANXA6 | 1.094775 | 7.567937 | 14.4387 | 1.54E-36 | 1.84E-33 |  |
| LOC100131043 | 1.094941 | 7.142416 | 11.32236 | 4.00E-25 | 3.00E-23 |  |
| DNAJB9 | 1.099051 | 8.463471 | 10.48051 | 3.24E-22 | 1.37E-20 |  |
| SEC11C | 1.100997 | 9.306756 | 10.49577 | 2.88E-22 | 1.24E-20 |  |
| MMP1 | 1.103133 | 6.79129 | 5.335216 | 1.84E-07 | 9.13E-07 | 3 |
| CXCL12 | 1.106414 | 10.12765 | 14.17811 | 1.48E-35 | 1.00E-32 | 2 |
| PPAPDC1B | 1.106611 | 8.002327 | 11.12483 | 1.96E-24 | 1.27E-22 |  |
| RCSD1 | 1.106886 | 6.367741 | 12.80791 | 1.84E-30 | 3.36E-28 |  |
| ST6GAL1 | 1.114448 | 6.084885 | 12.73954 | 3.27E-30 | 5.72E-28 |  |
| DUSP5 | 1.119281 | 8.782438 | 10.85113 | 1.75E-23 | 9.48E-22 |  |
| SERPINI1 | 1.119446 | 6.277787 | 11.33216 | 3.70E-25 | 2.79E-23 |  |
| IL2RG | 1.120057 | 8.01411 | 13.18533 | 7.49E-32 | 1.79E-29 |  |
| VCAN | 1.120217 | 9.365596 | 11.36749 | 2.78E-25 | 2.13E-23 |  |
| CD177 | 1.124261 | 8.689573 | 8.016936 | 2.20E-14 | 2.94E-13 |  |
| RGS4 | 1.124666 | 6.981112 | 11.10576 | 2.29E-24 | 1.46E-22 | 1 |
| PECAM1 | 1.125314 | 8.604853 | 16.49497 | 2.23E-44 | 4.53E-40 |  |
| CCL18 | 1.125751 | 7.922941 | 8.635978 | 3.07E-16 | 5.32E-15 |  |
| MMP3 | 1.128342 | 6.72827 | 5.709335 | 2.64E-08 | 1.50E-07 | 3 |
| HERPUD1 | 1.132315 | 11.14541 | 11.6348 | 3.16E-26 | 2.88E-24 |  |
| ZNF275 | 1.138967 | 7.491046 | 13.04936 | 2.38E-31 | 5.14E-29 |  |
| MMP13 | 1.139412 | 7.661986 | 7.000153 | 1.57E-11 | 1.40E-10 |  |
| RNASE6 | 1.139501 | 7.28831 | 12.34669 | 8.82E-29 | 1.27E-26 |  |
| EGFL6 | 1.149254 | 8.407624 | 12.36263 | 7.72E-29 | 1.12E-26 |  |
| C21orf96 | 1.150976 | 7.865837 | 14.08492 | 3.32E-35 | 2.04E-32 |  |
| ENPP2 | 1.156169 | 7.617204 | 12.88368 | 9.69E-31 | 1.87E-28 |  |
| CYR61 | 1.15844 | 8.675042 | 9.889651 | 3.09E-20 | 9.63E-19 |  |
| SEL1L3 | 1.164745 | 6.317821 | 13.37803 | 1.45E-32 | 3.92E-30 |  |
| LY96 | 1.16956 | 8.960122 | 11.71761 | 1.60E-26 | 1.53E-24 |  |
| TAGAP | 1.173264 | 6.553601 | 13.66898 | 1.20E-33 | 4.59E-31 |  |
| LOC100508797 | 1.186475 | 4.966209 | 13.1577 | 9.48E-32 | 2.19E-29 |  |
| BTG2 | 1.190949 | 9.056202 | 13.43221 | 9.11E-33 | 2.57E-30 |  |
| C3 | 1.199042 | 8.768186 | 14.42115 | 1.80E-36 | 2.03E-33 | 1 |
| COL4A1 | 1.204868 | 9.154918 | 14.5071 | 8.52E-37 | 1.08E-33 |  |
| IGKC | 1.205121 | 10.0923 | 13.15744 | 9.50E-32 | 2.19E-29 |  |
| RAC2 | 1.211323 | 7.930077 | 14.35775 | 3.12E-36 | 2.88E-33 | 1 |
| RGS1 | 1.212756 | 6.42007 | 10.62889 | 1.01E-22 | 4.76E-21 | 1 |
| POU2AF1 | 1.219795 | 6.398188 | 14.36925 | 2.82E-36 | 2.87E-33 |  |
| KCNN3 | 1.228816 | 6.377987 | 11.2792 | 5.67E-25 | 4.05E-23 |  |
| ITM2C | 1.235169 | 8.604388 | 12.07495 | 8.45E-28 | 1.02E-25 |  |
| IL8 | 1.235867 | 8.202008 | 8.103255 | 1.23E-14 | 1.71E-13 | 3 |
| COL15A1 | 1.242556 | 9.712492 | 15.42728 | 2.73E-40 | 1.38E-36 |  |
| CSF2RB | 1.248579 | 8.654263 | 14.52152 | 7.51E-37 | 1.02E-33 |  |
| P2RY8 | 1.254419 | 7.402325 | 11.94457 | 2.48E-27 | 2.67E-25 |  |
| COL4A2 | 1.25476 | 8.85516 | 12.76583 | 2.62E-30 | 4.67E-28 |  |
| NCF4 | 1.257569 | 6.92356 | 12.20461 | 2.88E-28 | 3.80E-26 | 1 |
| CD19 | 1.259514 | 6.822974 | 12.67103 | 5.82E-30 | 9.85E-28 | 2 |
| HSPA13 | 1.259898 | 7.885659 | 10.58974 | 1.38E-22 | 6.23E-21 |  |
| CSF3 | 1.260013 | 7.250765 | 12.02537 | 1.27E-27 | 1.47E-25 | 3 |
| PLAC8 | 1.262348 | 8.325107 | 9.318437 | 2.23E-18 | 5.29E-17 |  |
| SELM | 1.264609 | 8.664292 | 11.94913 | 2.39E-27 | 2.60E-25 |  |
| SELE | 1.27061 | 8.548202 | 10.44685 | 4.22E-22 | 1.74E-20 | 3 |
| HCLS1 | 1.27201 | 8.411898 | 14.78095 | 7.83E-38 | 1.59E-34 | 1 |
| IGLL3P | 1.280922 | 12.15574 | 14.759 | 9.48E-38 | 1.75E-34 |  |
| IGJ | 1.301333 | 10.15553 | 9.328087 | 2.07E-18 | 4.95E-17 |  |
| RHOH | 1.312124 | 6.690382 | 12.05979 | 9.58E-28 | 1.14E-25 |  |
| PDZRN4 | 1.312376 | 6.37295 | 10.81877 | 2.26E-23 | 1.19E-21 |  |
| FOS | 1.3227 | 10.26949 | 8.574493 | 4.73E-16 | 7.98E-15 | 3 |
| FCGR2B | 1.323876 | 7.56908 | 12.03698 | 1.16E-27 | 1.36E-25 | 3 |
| ANKRD36BP2 | 1.330522 | 6.049169 | 11.97292 | 1.96E-27 | 2.18E-25 |  |
| FCGR3B | 1.338999 | 7.160503 | 10.70533 | 5.54E-23 | 2.70E-21 |  |
| MME | 1.341307 | 6.957525 | 14.25164 | 7.83E-36 | 6.12E-33 |  |
| PIM2 | 1.35984 | 7.671532 | 12.09458 | 7.18E-28 | 8.84E-26 |  |
| ICAM3 | 1.364532 | 7.940134 | 12.15761 | 4.26E-28 | 5.54E-26 |  |
| IGLV4-60 | 1.372004 | 7.171933 | 9.491015 | 6.20E-19 | 1.58E-17 |  |
| ICAM2 | 1.386887 | 8.064677 | 14.67552 | 1.96E-37 | 2.85E-34 |  |
| C1orf38 | 1.395664 | 7.328388 | 13.90735 | 1.54E-34 | 7.81E-32 |  |
| IL10RA | 1.397464 | 7.951115 | 13.79426 | 4.08E-34 | 1.80E-31 | 1 |
| CXCL13 | 1.401202 | 10.1892 | 10.4843 | 3.15E-22 | 1.33E-20 | 1 |
| CYP24A1 | 1.407229 | 6.915951 | 12.86587 | 1.13E-30 | 2.10E-28 |  |
| ODAM | 1.421622 | 10.81133 | 9.435648 | 9.35E-19 | 2.32E-17 |  |
| PLAT | 1.451062 | 9.129979 | 14.31957 | 4.34E-36 | 3.84E-33 | 3 |
| SFRP4 | 1.454729 | 6.596634 | 11.18199 | 1.24E-24 | 8.37E-23 |  |
| FKBP11 | 1.489568 | 8.938791 | 13.64537 | 1.47E-33 | 5.51E-31 |  |
| PNOC | 1.491076 | 6.482068 | 11.61104 | 3.83E-26 | 3.43E-24 | 1 |
| MMP12 | 1.494215 | 9.618952 | 9.255086 | 3.55E-18 | 8.11E-17 |  |
| FCRLA | 1.497705 | 6.036518 | 10.99653 | 5.49E-24 | 3.29E-22 |  |
| SELL | 1.513505 | 6.918135 | 13.55812 | 3.10E-33 | 1.03E-30 | 3 |
| FCRL5 | 1.518107 | 6.174428 | 12.23605 | 2.22E-28 | 3.00E-26 |  |
| MMP7 | 1.536916 | 7.239429 | 11.47792 | 1.13E-25 | 9.44E-24 |  |
| EVI2B | 1.543661 | 8.091159 | 13.61105 | 1.97E-33 | 6.89E-31 | 1 |
| PTP4A3 | 1.616516 | 7.498264 | 12.8909 | 9.11E-31 | 1.78E-28 |  |
| C16orf54 | 1.623144 | 6.207117 | 11.4294 | 1.68E-25 | 1.36E-23 |  |
| CECR1 | 1.627598 | 8.596615 | 13.68249 | 1.07E-33 | 4.42E-31 |  |
| CD38 | 1.633634 | 7.579309 | 12.40691 | 5.33E-29 | 7.99E-27 | 2 |
| IGKV1D-8 | 1.671809 | 6.979288 | 11.06747 | 3.11E-24 | 1.96E-22 |  |
| IGLC1 | 1.684645 | 7.803135 | 12.54295 | 1.71E-29 | 2.69E-27 |  |
| CPNE5 | 1.685516 | 6.75212 | 13.31236 | 2.54E-32 | 6.61E-30 |  |
| CYTIP | 1.704076 | 7.313038 | 13.14747 | 1.03E-31 | 2.33E-29 | 2 |
| SLAMF7 | 1.715182 | 7.715118 | 11.97088 | 2.00E-27 | 2.20E-25 |  |
| CHST2 | 1.744351 | 8.078286 | 14.83083 | 5.07E-38 | 1.14E-34 |  |
| LAX1 | 1.74535 | 7.648284 | 12.38772 | 6.26E-29 | 9.22E-27 |  |
| CXCR4 | 1.814783 | 8.284652 | 15.362 | 4.84E-40 | 1.96E-36 | 2 |
| CD27 | 1.874988 | 8.241171 | 14.1885 | 1.35E-35 | 9.48E-33 | 2 |
| CXCL1 | 1.895931 | 10.13333 | 14.1529 | 1.84E-35 | 1.21E-32 | 1 |
| IGHM | 1.997567 | 8.780853 | 13.8893 | 1.80E-34 | 8.90E-32 |  |
| CXCL6 | 2.036943 | 7.718308 | 11.77012 | 1.04E-26 | 1.02E-24 | 1 |
| CD79A | 2.039516 | 8.246125 | 13.4894 | 5.59E-33 | 1.72E-30 | 2 |
| IGKV1-5 | 2.044175 | 8.517703 | 13.77604 | 4.77E-34 | 2.06E-31 |  |
| FAM46C | 2.092727 | 8.462554 | 14.71583 | 1.38E-37 | 2.16E-34 |  |
| IGLV3-19 | 2.196797 | 9.161593 | 13.55093 | 3.30E-33 | 1.08E-30 |  |
| SPAG4 | 2.205266 | 7.406673 | 13.43747 | 8.71E-33 | 2.51E-30 |  |
| IGLV1-40 | 2.218375 | 9.05981 | 13.83521 | 2.86E-34 | 1.35E-31 |  |
| KIAA0125 | 2.268105 | 7.670168 | 12.67475 | 5.64E-30 | 9.63E-28 |  |
| IGKV3-20 | 2.312742 | 9.260228 | 14.96094 | 1.62E-38 | 5.50E-35 |  |
| IGLV2-23 | 2.318765 | 11.41593 | 14.40189 | 2.13E-36 | 2.27E-33 |  |
| IGLJ3 | 2.420145 | 10.6425 | 14.93537 | 2.03E-38 | 5.89E-35 |  |
| TNFRSF17 | 2.462987 | 7.938006 | 12.97812 | 4.35E-31 | 9.12E-29 |  |
| MGC29506 | 2.492085 | 9.365121 | 14.22629 | 9.76E-36 | 7.34E-33 |  |
| IGLV1-36 | 2.593034 | 10.78688 | 13.57441 | 2.70E-33 | 9.12E-31 |  |
| IGKV4-1 | 2.617356 | 11.40801 | 13.43657 | 8.78E-33 | 2.51E-30 |  |
| DSC1 | -2.21452 | 6.26258 | -10.8871 | 1.31E-23 | 7.36E-22 |  |
| AADACL2 | -2.07064 | 4.977933 | -11.6236 | 3.46E-26 | 3.14E-24 |  |
| FLG2 | -1.74245 | 5.328664 | -9.0437 | 1.66E-17 | 3.44E-16 |  |
| KRT2 | -1.71052 | 6.82402 | -6.96434 | 1.96E-11 | 1.72E-10 |  |
| ELMOD1 | -1.44676 | 4.519634 | -11.1575 | 1.51E-24 | 9.96E-23 |  |
| LOR | -1.37592 | 8.931259 | -6.27838 | 1.15E-09 | 7.93E-09 |  |
| BPIL2 | -1.32609 | 7.596343 | -12.4477 | 3.79E-29 | 5.84E-27 |  |
| ELOVL4 | -1.30576 | 7.521901 | -11.6629 | 2.51E-26 | 2.31E-24 |  |
| CALML5 | -1.30092 | 7.479515 | -8.9087 | 4.39E-17 | 8.58E-16 |  |
| FLG | -1.30081 | 7.571284 | -7.32693 | 2.03E-12 | 2.06E-11 |  |
| CLDN20 | -1.29595 | 4.766157 | -13.4547 | 7.52E-33 | 2.21E-30 |  |
| NEFL | -1.2628 | 6.053856 | -12.922 | 7.00E-31 | 1.41E-28 |  |
| AADAC | -1.25726 | 5.695609 | -11.6734 | 2.30E-26 | 2.13E-24 |  |
| C5orf23 | -1.25665 | 7.218103 | -11.8812 | 4.18E-27 | 4.31E-25 |  |
| SLC27A6 | -1.24873 | 4.866944 | -10.2253 | 2.36E-21 | 8.62E-20 |  |
| ATP6V1C2 | -1.18728 | 6.03148 | -13.6223 | 1.79E-33 | 6.48E-31 |  |
| ABCA12 | -1.16317 | 6.568385 | -9.66715 | 1.66E-19 | 4.61E-18 |  |
| LCE2B | -1.14777 | 5.262836 | -8.76788 | 1.20E-16 | 2.21E-15 |  |
| MAMDC2 | -1.13288 | 5.568723 | -8.0027 | 2.42E-14 | 3.22E-13 |  |
| SLC16A9 | -1.11711 | 8.714972 | -11.4967 | 9.74E-26 | 8.22E-24 |  |
| RPTN | -1.09805 | 10.2027 | -6.30551 | 9.82E-10 | 6.84E-09 |  |
| EPCAM | -1.0953 | 5.754301 | -10.1315 | 4.86E-21 | 1.71E-19 |  |
| KRT1 | -1.09494 | 11.50372 | -5.09011 | 6.20E-07 | 2.83E-06 |  |
| POF1B | -1.07848 | 6.003318 | -12.3293 | 1.02E-28 | 1.45E-26 |  |
| SLC19A2 | -1.06538 | 6.926333 | -11.3749 | 2.62E-25 | 2.02E-23 |  |
| NPR3 | -1.06419 | 4.943889 | -13.3518 | 1.81E-32 | 4.78E-30 |  |
| CDSN | -1.05989 | 7.525609 | -6.69153 | 1.03E-10 | 8.15E-10 |  |
| ASPRV1 | -1.05212 | 7.384341 | -6.35553 | 7.37E-10 | 5.23E-09 |  |
| FAM83C | -1.0513 | 7.0764 | -12.9497 | 5.54E-31 | 1.15E-28 |  |
| LY6G6C | -1.04436 | 8.050037 | -8.78924 | 1.03E-16 | 1.91E-15 |  |
| CYP3A5 | -1.04215 | 7.141287 | -10.5781 | 1.51E-22 | 6.81E-21 |  |
| LCE1B | -1.03311 | 5.585756 | -9.01662 | 2.02E-17 | 4.13E-16 |  |
| COBL | -1.01972 | 5.747029 | -13.6193 | 1.83E-33 | 6.53E-31 |  |
| YOD1 | -1.01333 | 7.076793 | -10.3473 | 9.15E-22 | 3.58E-20 |  |
| CAPNS2 | -1.01095 | 9.263439 | -11.3418 | 3.42E-25 | 2.60E-23 |  |
| WFDC5 | -0.99914 | 6.911419 | -10.0471 | 9.29E-21 | 3.12E-19 |  |
| ANKRD29 | -0.99866 | 6.148876 | -11.9102 | 3.30E-27 | 3.43E-25 |  |
| CYP4F22 | -0.99453 | 6.548399 | -10.4288 | 4.85E-22 | 1.98E-20 |  |
| HLF | -0.99437 | 6.915706 | -10.6264 | 1.03E-22 | 4.83E-21 |  |
| MAP2 | -0.98306 | 6.511337 | -12.8926 | 8.98E-31 | 1.77E-28 |  |
| MUC15 | -0.97522 | 8.180699 | -9.81358 | 5.50E-20 | 1.65E-18 |  |
| DNER | -0.96881 | 3.61343 | -8.71794 | 1.72E-16 | 3.07E-15 |  |
| HPGD | -0.96845 | 7.30572 | -9.25357 | 3.59E-18 | 8.18E-17 |  |
| AHNAK2 | -0.95813 | 8.221816 | -13.6736 | 1.15E-33 | 4.57E-31 |  |
| DAPL1 | -0.95076 | 9.82332 | -9.51085 | 5.35E-19 | 1.38E-17 |  |
| FAM57A | -0.94917 | 7.705107 | -13.8572 | 2.37E-34 | 1.15E-31 |  |
| CWH43 | -0.94615 | 7.822747 | -12.0363 | 1.16E-27 | 1.36E-25 |  |
| ANKRD35 | -0.94495 | 7.57342 | -11.9816 | 1.83E-27 | 2.05E-25 |  |
| LRIG3 | -0.92729 | 7.0179 | -9.16834 | 6.69E-18 | 1.48E-16 |  |
| KIF21A | -0.91168 | 6.305392 | -13.482 | 5.95E-33 | 1.80E-30 |  |
| TPPP3 | -0.91105 | 7.166132 | -7.99999 | 2.47E-14 | 3.27E-13 |  |
| ALOX12B | -0.90647 | 7.691959 | -7.85115 | 6.69E-14 | 8.25E-13 |  |
| ISL1 | -0.90573 | 4.176845 | -11.4719 | 1.19E-25 | 9.87E-24 |  |
| ZNF57 | -0.90289 | 6.3235 | -11.6037 | 4.07E-26 | 3.61E-24 |  |
| ARRDC4 | -0.90198 | 7.439349 | -9.68233 | 1.48E-19 | 4.14E-18 |  |
| STK39 | -0.90166 | 7.462863 | -10.8429 | 1.86E-23 | 9.99E-22 |  |
| KRT10 | -0.90032 | 11.48355 | -7.64911 | 2.54E-13 | 2.89E-12 |  |
| HS3ST6 | -0.89611 | 4.98288 | -10.8451 | 1.83E-23 | 9.89E-22 |  |
| GSTA4 | -0.89507 | 8.490416 | -11.1559 | 1.53E-24 | 1.01E-22 |  |
| TYRP1 | -0.89299 | 5.633696 | -3.62492 | 0.000337 | 0.000965 |  |
| DSG1 | -0.89005 | 11.00063 | -7.30996 | 2.26E-12 | 2.28E-11 |  |
| D4S234E | -0.88869 | 8.139445 | -12.8948 | 8.81E-31 | 1.75E-28 |  |
| CYP2C18 | -0.88562 | 8.015543 | -11.8269 | 6.54E-27 | 6.64E-25 |  |
| KRT80 | -0.8735 | 8.68023 | -11.0277 | 4.28E-24 | 2.60E-22 |  |
| BBOX1 | -0.86555 | 8.304276 | -9.86925 | 3.61E-20 | 1.11E-18 |  |
| ENDOU | -0.86125 | 8.811534 | -8.95069 | 3.25E-17 | 6.46E-16 |  |
| KRT3 | -0.84858 | 6.282678 | -5.39567 | 1.35E-07 | 6.86E-07 |  |
| STXBP5 | -0.84178 | 6.010072 | -10.5465 | 1.93E-22 | 8.57E-21 |  |
| OSBPL6 | -0.83918 | 5.375705 | -14.0429 | 4.77E-35 | 2.85E-32 |  |
| BNIPL | -0.83521 | 7.139046 | -11.688 | 2.04E-26 | 1.92E-24 |  |
| HSPC159 | -0.8322 | 9.293576 | -12.2951 | 1.36E-28 | 1.89E-26 |  |
| GRAMD1C | -0.82321 | 6.486249 | -7.68754 | 1.97E-13 | 2.29E-12 |  |
| STX19 | -0.82194 | 4.465666 | -12.6206 | 8.90E-30 | 1.43E-27 |  |
| ANKRD6 | -0.8217 | 6.540286 | -12.1695 | 3.86E-28 | 5.05E-26 |  |
| LOC344887 | -0.8182 | 6.11965 | -8.20349 | 6.20E-15 | 8.96E-14 |  |
| BAMBI | -0.81466 | 6.931648 | -8.92289 | 3.97E-17 | 7.81E-16 |  |
| CBR3 | -0.81157 | 8.877204 | -10.7392 | 4.24E-23 | 2.13E-21 |  |
| HMGCR | -0.80684 | 8.576978 | -10.9961 | 5.51E-24 | 3.29E-22 |  |
| ZNF750 | -0.80477 | 9.952137 | -9.86132 | 3.83E-20 | 1.18E-18 |  |
| ADH7 | -0.80454 | 7.99196 | -6.61143 | 1.65E-10 | 1.28E-09 |  |
| GGH | -0.80029 | 8.609915 | -11.721 | 1.56E-26 | 1.50E-24 |  |
| ZNF823 | -0.79746 | 6.227535 | -9.82423 | 5.07E-20 | 1.53E-18 |  |
| LOC100506828 | -0.79604 | 5.451524 | -8.08997 | 1.34E-14 | 1.86E-13 |  |
| CXCL14 | -0.79515 | 9.269061 | -9.43724 | 9.24E-19 | 2.29E-17 |  |
| SERPINB7 | -0.78768 | 5.677996 | -8.57626 | 4.67E-16 | 7.90E-15 |  |
| FAM3B | -0.78607 | 8.036114 | -8.80864 | 9.00E-17 | 1.69E-15 |  |
| LOC100134445 | -0.78235 | 7.131294 | -6.61288 | 1.64E-10 | 1.27E-09 |  |
| SDR9C7 | -0.77659 | 7.136967 | -7.03091 | 1.30E-11 | 1.17E-10 |  |
| PANK1 | -0.77608 | 5.973439 | -8.85823 | 6.31E-17 | 1.21E-15 |  |
| PLXDC2 | -0.77572 | 7.240483 | -13.5039 | 4.93E-33 | 1.57E-30 |  |
| DYNLT3 | -0.77366 | 10.40366 | -10.6142 | 1.14E-22 | 5.29E-21 |  |
| FZD10 | -0.77332 | 7.825391 | -8.64587 | 2.86E-16 | 4.98E-15 |  |
| CHRNA9 | -0.77268 | 4.641734 | -8.13958 | 9.59E-15 | 1.35E-13 |  |
| TMEM79 | -0.77183 | 9.179113 | -10.1826 | 3.28E-21 | 1.18E-19 |  |
| PAQR5 | -0.77166 | 5.782489 | -11.5672 | 5.48E-26 | 4.82E-24 |  |
| SPAG17 | -0.76901 | 4.785276 | -13.9866 | 7.76E-35 | 4.38E-32 |  |
| LOC283404 | -0.7654 | 6.212875 | -10.5325 | 2.16E-22 | 9.48E-21 |  |
| EXPH5 | -0.76538 | 6.922069 | -10.299 | 1.33E-21 | 5.04E-20 |  |
| TGM7 | -0.76435 | 6.095892 | -8.78598 | 1.06E-16 | 1.95E-15 |  |
| LCE3D | -0.76406 | 10.92067 | -6.13205 | 2.62E-09 | 1.73E-08 |  |
| PPP1R14C | -0.76405 | 8.06367 | -12.771 | 2.51E-30 | 4.51E-28 |  |
| TUFT1 | -0.75999 | 8.649639 | -10.639 | 9.35E-23 | 4.41E-21 |  |
| IFFO2 | -0.75828 | 9.171136 | -9.63999 | 2.04E-19 | 5.59E-18 |  |
| PP13439 | -0.75535 | 5.174612 | -8.57337 | 4.76E-16 | 8.04E-15 |  |
| GRHL3 | -0.74958 | 8.93305 | -11.2557 | 6.86E-25 | 4.83E-23 |  |
| DSC2 | -0.74463 | 10.81105 | -9.7273 | 1.06E-19 | 3.03E-18 |  |
| LOC100507455 | -0.73867 | 7.243514 | -9.44947 | 8.44E-19 | 2.11E-17 |  |
| SLURP1 | -0.73667 | 9.006473 | -6.901 | 2.89E-11 | 2.48E-10 |  |
| LOC284837 | -0.73609 | 5.636265 | -9.27298 | 3.11E-18 | 7.19E-17 |  |
| ARL9 | -0.73603 | 6.162188 | -13.1952 | 6.89E-32 | 1.69E-29 |  |
| ZNF273 | -0.73183 | 5.900699 | -9.36859 | 1.54E-18 | 3.71E-17 |  |
| CDCA7 | -0.73158 | 5.434667 | -10.0222 | 1.12E-20 | 3.73E-19 |  |
| FAM59A | -0.7306 | 5.723846 | -10.0483 | 9.20E-21 | 3.10E-19 |  |
| H19 | -0.72865 | 6.651662 | -4.9287 | 1.35E-06 | 5.80E-06 |  |
| A2ML1 | -0.72832 | 8.517864 | -8.31266 | 2.93E-15 | 4.43E-14 |  |
| NEFM | -0.72721 | 5.239404 | -13.813 | 3.47E-34 | 1.57E-31 |  |
| HOOK1 | -0.72341 | 7.617337 | -9.98452 | 1.50E-20 | 4.88E-19 |  |
| LOC284219 | -0.72 | 6.458275 | -8.83221 | 7.60E-17 | 1.45E-15 |  |
| SLC47A2 | -0.71961 | 6.801911 | -7.14866 | 6.26E-12 | 5.89E-11 |  |
| HRASLS | -0.71743 | 5.223988 | -9.62271 | 2.32E-19 | 6.33E-18 |  |
| GDA | -0.71569 | 4.996291 | -8.98454 | 2.54E-17 | 5.14E-16 |  |
| PSORS1C2 | -0.71518 | 5.809833 | -6.71515 | 8.90E-11 | 7.14E-10 |  |
| DEGS1 | -0.71489 | 9.434548 | -10.9295 | 9.37E-24 | 5.37E-22 |  |
| DEFB1 | -0.71452 | 7.158575 | -7.72195 | 1.57E-13 | 1.85E-12 |  |
| LOC653602 | -0.71441 | 5.76372 | -6.43781 | 4.58E-10 | 3.33E-09 |  |
| XK | -0.71347 | 5.683209 | -7.75316 | 1.28E-13 | 1.52E-12 |  |
| KIAA1598 | -0.7131 | 7.752232 | -9.31397 | 2.30E-18 | 5.44E-17 |  |
| VAV3 | -0.70939 | 7.464078 | -7.00245 | 1.55E-11 | 1.38E-10 |  |
| DMKN | -0.70879 | 9.234469 | -7.20044 | 4.52E-12 | 4.36E-11 |  |
| ANK3 | -0.70573 | 6.756134 | -13.3755 | 1.48E-32 | 3.95E-30 |  |
| EEA1 | -0.70273 | 6.302304 | -11.0292 | 4.23E-24 | 2.58E-22 |  |
| EPHX3 | -0.70187 | 8.358672 | -9.53904 | 4.33E-19 | 1.14E-17 |  |
| C6orf132 | -0.69958 | 7.821614 | -8.5391 | 6.06E-16 | 1.01E-14 |  |
| ALS2CR4 | -0.6978 | 6.434276 | -10.9184 | 1.02E-23 | 5.83E-22 |  |
| EPN3 | -0.69463 | 7.120962 | -12.1068 | 6.49E-28 | 8.18E-26 |  |
| BSPRY | -0.69438 | 7.445318 | -13.1312 | 1.19E-31 | 2.62E-29 |  |
| MAF | -0.69438 | 6.502562 | -11.9762 | 1.91E-27 | 2.13E-25 |  |
| CYP27C1 | -0.69301 | 4.315145 | -8.62914 | 3.22E-16 | 5.57E-15 |  |
| PIR | -0.6908 | 7.297954 | -8.12637 | 1.05E-14 | 1.47E-13 |  |
| TRPS1 | -0.68797 | 7.84782 | -10.5649 | 1.67E-22 | 7.52E-21 |  |
| TMEM14A | -0.68661 | 8.988487 | -10.0721 | 7.66E-21 | 2.62E-19 |  |
| AGPAT9 | -0.68308 | 6.399981 | -8.55013 | 5.61E-16 | 9.34E-15 |  |
| IL18 | -0.67994 | 8.064462 | -7.67744 | 2.11E-13 | 2.43E-12 |  |
| C12orf29 | -0.67964 | 8.133555 | -10.1507 | 4.19E-21 | 1.49E-19 |  |
| DLX3 | -0.67927 | 6.810929 | -7.1506 | 6.18E-12 | 5.83E-11 |  |
| IFI27 | -0.67892 | 10.18225 | -5.36414 | 1.59E-07 | 7.99E-07 |  |
| SSX2IP | -0.67527 | 5.715615 | -11.8606 | 4.95E-27 | 5.08E-25 |  |
| IL7 | -0.67508 | 5.195478 | -7.18393 | 5.02E-12 | 4.82E-11 | 2 |
| RORA | -0.67448 | 5.650599 | -13.9498 | 1.07E-34 | 5.83E-32 |  |
| BNIP3 | -0.67319 | 8.350293 | -10.8429 | 1.86E-23 | 9.99E-22 |  |
| C10orf99 | -0.67309 | 10.44386 | -8.28536 | 3.53E-15 | 5.28E-14 |  |
| ODZ2 | -0.67058 | 7.826831 | -9.08581 | 1.22E-17 | 2.58E-16 |  |
| GGT6 | -0.66926 | 6.653373 | -10.0945 | 6.46E-21 | 2.24E-19 |  |
| SERPINB11 | -0.66864 | 8.536746 | -3.1756 | 0.001645 | 0.004113 |  |
| SOCS6 | -0.66827 | 6.146497 | -8.8951 | 4.84E-17 | 9.39E-16 |  |
| LANCL3 | -0.66551 | 4.533867 | -8.44165 | 1.20E-15 | 1.93E-14 |  |
| ELF5 | -0.66469 | 6.032514 | -7.13356 | 6.88E-12 | 6.43E-11 |  |
| CD36 | -0.66428 | 5.496836 | -8.75991 | 1.27E-16 | 2.32E-15 |  |
| E2F8 | -0.66332 | 6.027119 | -7.99878 | 2.49E-14 | 3.29E-13 |  |
| C11orf45 | -0.66224 | 4.844947 | -9.61774 | 2.41E-19 | 6.56E-18 |  |
| KLK7 | -0.65902 | 9.950163 | -8.70077 | 1.94E-16 | 3.44E-15 |  |
| FAM83B | -0.65732 | 5.216896 | -6.8638 | 3.63E-11 | 3.07E-10 |  |
| GBP6 | -0.65711 | 9.068765 | -8.2894 | 3.44E-15 | 5.15E-14 |  |
| IRX4 | -0.65626 | 6.183476 | -9.49145 | 6.18E-19 | 1.58E-17 |  |
| OCLN | -0.65621 | 6.59515 | -10.8673 | 1.54E-23 | 8.45E-22 |  |
| GPR115 | -0.65552 | 5.218475 | -10.8686 | 1.52E-23 | 8.40E-22 |  |
| DCT | -0.65546 | 4.899865 | -6.47205 | 3.75E-10 | 2.75E-09 |  |
| SLK | -0.65519 | 8.9321 | -9.15313 | 7.48E-18 | 1.64E-16 |  |
| HTR3A | -0.65432 | 5.812641 | -7.14039 | 6.59E-12 | 6.18E-11 |  |
| ACPP | -0.6537 | 6.524068 | -9.87335 | 3.50E-20 | 1.09E-18 |  |
| C6orf142 | -0.65311 | 5.18035 | -9.15204 | 7.54E-18 | 1.65E-16 |  |
| ABLIM1 | -0.65098 | 9.391788 | -9.51588 | 5.15E-19 | 1.33E-17 |  |
| KLF4 | -0.65046 | 10.26216 | -7.75914 | 1.23E-13 | 1.47E-12 |  |
| DSC3 | -0.6476 | 10.53138 | -9.19984 | 5.32E-18 | 1.19E-16 |  |
| PLEKHA1 | -0.64714 | 8.75699 | -10.2684 | 1.69E-21 | 6.27E-20 |  |
| NRTN | -0.64385 | 4.04953 | -9.7737 | 7.44E-20 | 2.19E-18 |  |
| KRT23 | -0.64333 | 7.385941 | -5.40504 | 1.29E-07 | 6.56E-07 |  |
| FAM110C | -0.64307 | 9.014296 | -7.87292 | 5.79E-14 | 7.21E-13 |  |
| SLC16A6 | -0.64237 | 7.191766 | -9.6018 | 2.71E-19 | 7.36E-18 |  |
| USP53 | -0.64157 | 5.736769 | -10.8002 | 2.62E-23 | 1.36E-21 |  |
| ZNRF3 | -0.64138 | 6.455071 | -8.18744 | 6.92E-15 | 9.94E-14 |  |
| C7orf57 | -0.6406 | 3.304747 | -10.6406 | 9.23E-23 | 4.37E-21 |  |
| TMEM117 | -0.64057 | 7.440778 | -10.0502 | 9.07E-21 | 3.06E-19 |  |
| ABHD5 | -0.63954 | 6.384196 | -12.1059 | 6.54E-28 | 8.20E-26 |  |
| BNC1 | -0.63922 | 7.173864 | -9.93346 | 2.21E-20 | 7.02E-19 |  |
| HORMAD1 | -0.6387 | 4.207971 | -4.21107 | 3.33E-05 | 0.000114 |  |
| CSRP2 | -0.6383 | 9.294089 | -9.45774 | 7.94E-19 | 2.00E-17 |  |
| TGM5 | -0.63803 | 7.763058 | -9.39498 | 1.26E-18 | 3.07E-17 |  |
| ALOXE3 | -0.63662 | 5.520986 | -8.73869 | 1.48E-16 | 2.68E-15 |  |
| DUSP14 | -0.6355 | 9.102071 | -11.1209 | 2.03E-24 | 1.30E-22 |  |
| SPRR2G | -0.63547 | 10.71548 | -4.3218 | 2.08E-05 | 7.39E-05 |  |
| ZHX1 | -0.6347 | 7.301395 | -8.01259 | 2.27E-14 | 3.02E-13 |  |
| MLF1IP | -0.63391 | 6.09104 | -9.29273 | 2.69E-18 | 6.30E-17 |  |
| KLK10 | -0.63312 | 9.56891 | -8.22853 | 5.22E-15 | 7.64E-14 |  |
| CLN8 | -0.63222 | 6.556633 | -13.1443 | 1.06E-31 | 2.37E-29 |  |
| TNFRSF19 | -0.63028 | 5.850682 | -8.76249 | 1.25E-16 | 2.29E-15 |  |
| FBXO45 | -0.62975 | 6.792174 | -12.1017 | 6.77E-28 | 8.39E-26 |  |
| GPSM2 | -0.6274 | 7.074499 | -10.5411 | 2.02E-22 | 8.92E-21 |  |
| TXN | -0.62603 | 9.417334 | -8.39797 | 1.62E-15 | 2.54E-14 |  |
| KIAA1737 | -0.62542 | 6.449971 | -8.67436 | 2.34E-16 | 4.12E-15 |  |
| C5orf46 | -0.62465 | 5.050532 | -8.68336 | 2.19E-16 | 3.88E-15 |  |
| DENND2C | -0.61951 | 7.103803 | -12.3146 | 1.15E-28 | 1.61E-26 |  |
| IMPA2 | -0.61862 | 9.294842 | -7.74693 | 1.34E-13 | 1.58E-12 |  |
| ESYT3 | -0.61798 | 4.243661 | -11.8862 | 4.01E-27 | 4.16E-25 |  |
| FAM83G | -0.61766 | 7.459884 | -9.95947 | 1.81E-20 | 5.85E-19 |  |
| UNG | -0.61757 | 7.049421 | -10.2036 | 2.79E-21 | 1.01E-19 |  |
| BDNF | -0.61708 | 4.437806 | -13.0339 | 2.71E-31 | 5.80E-29 |  |
| MEIS1 | -0.61655 | 6.904966 | -8.6541 | 2.70E-16 | 4.72E-15 |  |
| RAPH1 | -0.61459 | 6.492032 | -9.73709 | 9.81E-20 | 2.83E-18 |  |
| PCDHB14 | -0.61456 | 4.985053 | -6.91738 | 2.62E-11 | 2.26E-10 |  |
| TRIM7 | -0.61194 | 6.581242 | -12.213 | 2.69E-28 | 3.56E-26 |  |
| FAHD1 | -0.61141 | 6.945748 | -11.3751 | 2.61E-25 | 2.02E-23 |  |
| PTPRZ1 | -0.61114 | 7.54346 | -6.75754 | 6.90E-11 | 5.62E-10 |  |
| KRT76 | -0.60896 | 12.91907 | -4.20292 | 3.45E-05 | 0.000118 |  |
| CHP2 | -0.60743 | 5.004823 | -10.4796 | 3.26E-22 | 1.37E-20 |  |
| SLC16A7 | -0.60652 | 5.546196 | -7.97178 | 2.99E-14 | 3.88E-13 |  |
| CD207 | -0.60621 | 5.696214 | -7.95161 | 3.42E-14 | 4.38E-13 |  |
| MATN2 | -0.60617 | 7.693556 | -8.49219 | 8.42E-16 | 1.38E-14 |  |
| EAF1 | -0.60548 | 6.819639 | -9.35904 | 1.65E-18 | 3.97E-17 |  |
| LRP4 | -0.6052 | 6.106555 | -9.33989 | 1.90E-18 | 4.54E-17 |  |
| ZNF658 | -0.60504 | 4.841315 | -6.85186 | 3.90E-11 | 3.29E-10 |  |
| TUBB2A | -0.6048 | 11.47657 | -10.4822 | 3.20E-22 | 1.35E-20 |  |
| SLC25A43 | -0.60477 | 7.837298 | -7.9153 | 4.36E-14 | 5.52E-13 |  |
| ANKRD57 | -0.6044 | 9.652208 | -9.13665 | 8.44E-18 | 1.83E-16 |  |
| NEBL | -0.60426 | 6.870807 | -11.4915 | 1.02E-25 | 8.49E-24 |  |
| ACER1 | -0.60423 | 5.678614 | -10.5476 | 1.92E-22 | 8.52E-21 |  |
| C1orf46 | -0.60332 | 4.616775 | -10.1687 | 3.65E-21 | 1.30E-19 |  |
| FOXN1 | -0.60164 | 7.299928 | -8.51316 | 7.27E-16 | 1.20E-14 |  |
| COMP | -0.59979 | 6.503473 | -3.89977 | 0.000118 | 0.000369 |  |
| TP53AIP1 | -0.59971 | 6.651147 | -10.9664 | 6.98E-24 | 4.09E-22 |  |
| ZNF430 | -0.59863 | 6.151369 | -9.55203 | 3.93E-19 | 1.05E-17 |  |
| KLHL21 | -0.59713 | 7.202847 | -12.1015 | 6.78E-28 | 8.39E-26 |  |
| PIGW | -0.59635 | 6.371748 | -9.2913 | 2.72E-18 | 6.34E-17 |  |
| FLRT3 | -0.59613 | 4.812769 | -7.80179 | 9.29E-14 | 1.12E-12 |  |
| CDCA7L | -0.5948 | 7.064044 | -9.24453 | 3.83E-18 | 8.68E-17 |  |
| BPGM | -0.59476 | 7.793434 | -7.73439 | 1.45E-13 | 1.71E-12 |  |
| ESF1 | -0.59439 | 6.080517 | -8.63799 | 3.02E-16 | 5.25E-15 |  |
| TOM1L1 | -0.59382 | 6.640486 | -11.0547 | 3.45E-24 | 2.15E-22 |  |
| GJA3 | -0.59352 | 5.327926 | -12.5146 | 2.17E-29 | 3.38E-27 |  |
| WDR47 | -0.58993 | 8.047898 | -7.42612 | 1.08E-12 | 1.14E-11 |  |
| KCNK7 | -0.58805 | 5.797401 | -10.6071 | 1.20E-22 | 5.55E-21 |  |
| ESRP1 | -0.5868 | 9.282767 | -10.8638 | 1.58E-23 | 8.65E-22 |  |
| RGS20 | -0.58676 | 5.846206 | -12.118 | 5.91E-28 | 7.64E-26 | 1 |
| PGAP1 | -0.58644 | 5.233992 | -8.45811 | 1.07E-15 | 1.73E-14 |  |
| TMEM159 | -0.5839 | 6.246488 | -7.31484 | 2.19E-12 | 2.22E-11 |  |
| CTNNBIP1 | -0.58349 | 8.234496 | -9.82152 | 5.18E-20 | 1.56E-18 |  |
| LOC100289049 | -0.58321 | 5.189247 | -7.34939 | 1.76E-12 | 1.80E-11 |  |
| CNTNAP3 | -0.582 | 4.700656 | -7.81278 | 8.63E-14 | 1.05E-12 |  |
| EML1 | -0.5819 | 6.070623 | -8.45116 | 1.12E-15 | 1.81E-14 |  |
| NIP7 | -0.58185 | 7.288715 | -9.75591 | 8.51E-20 | 2.49E-18 |  |
| ELOVL7 | -0.58182 | 8.11403 | -8.40052 | 1.59E-15 | 2.51E-14 |  |
| LOC286052 | -0.58172 | 6.800746 | -8.39247 | 1.69E-15 | 2.63E-14 |  |
| LOC100506556 | -0.58118 | 7.412773 | -8.39813 | 1.62E-15 | 2.54E-14 |  |
| MFHAS1 | -0.58058 | 7.622702 | -10.4418 | 4.39E-22 | 1.81E-20 |  |
| MICALCL | -0.58046 | 5.00544 | -8.81074 | 8.86E-17 | 1.66E-15 |  |

FC, fold change; AveExpr, average expression value; FDR, false discovery rate.
